# Metal-Free Synthesis of α-H Chlorine Alkylaromatic Hydrocarbons Driven by Visible Light

**DOI:** 10.3390/molecules30020312

**Published:** 2025-01-14

**Authors:** Lidia De Luca, Luca Ledda, Andrea Porcheddu, Massimo Carraro, Luisa Pisano, Silvia Gaspa

**Affiliations:** 1 Dipartimento di Scienze Chimiche, Fisiche, Matematiche e Naturali, Università degli Studi di Sassari, Via Vienna 2, 07100 Sassari, Italy; ldeluca@uniss.it (L.D.L.); lucaledda@gmail.com (L.L.); mcarraro@uniss.it (M.C.); luisa@uniss.it (L.P.); 2 Dipartimento di Scienze Chimiche e Geologiche, Università degli Studi di Cagliari, Cittadella Universitaria, 09042 Monserrato, Italy; porcheddu@unica.it

**Keywords:** benzyl chloride, visible light, toluene

## Abstract

Chlorination is a widely used strategy at the industrial level. Chlorinated products represent indispensable building blocks in synthetic chemistry. Here, we report the synthesis of benzyl chlorides and α-chloro alkyl arenes, mediated by visible light, starting from variously substituted toluenes and *N,N*-dichloroacetamide as a chlorinating reagent. This methodology is a valid alternative to the syntheses previously reported in the literature. It is a metal-free process and does not involve the use of additives or radical initiators.

## 1. Introduction

Organochlorine compounds are among the most abundant compounds in nature and can be found in a wide range of classes of biomolecules, such as alkaloids, terpenoids and steroids [1]. Furthermore, organochlorines represent versatile building blocks in chemical synthesis and are key components in a multitude of functional materials and pharmaceutical active ingredients [2,3,4,5,6,7].

For these reasons, an important goal in chemical synthesis is the development of new, efficient synthetic strategies that allow site-selective chlorination in a specific C–H bond in an organic compound [5,6,8,9].

C–H bond halogenation is of great relevance as it is an essential process for the preparation of precursors for synthetic chemistry, e.g., the industrial synthesis of amphetamine-class drugs, artificial resins, dyes and photographic developers [7].

The main industrial approach for the preparation of benzyl chloride consists of the homolysis of elemental chlorine, activated by thermal or UV irradiation, where one hydrogen atom of toluene is substituted by one chlorine atom [10]. Chlorination proceeds with the formation of benzyl chloride along with undesirable poly-chlorination products, such as benzal chloride, benzotrichloride and chlorotoluene produced by the chlorination of the aromatic ring. Another methodology that is widely used at an industrial level is the reaction of benzene with paraformaldehyde, gaseous hydrochloric acid and anhydrous zinc chloride, which leads to the chloromethylation of benzene [7,11,12]. These synthetic procedures are widely used but have significant drawbacks related to the use of dangerous and difficult-to-handle reagents, complex reaction conditions and low conversion and yields of benzyl chloride due to the low selectivity of the process. Furthermore, not only is chlorine a very hazardous and corrosive chemical, but the theoretical limit for maximum chlorine usage is only 50%, since half of the dichlorine forms HCl as a side product.

**Scheme 1:**
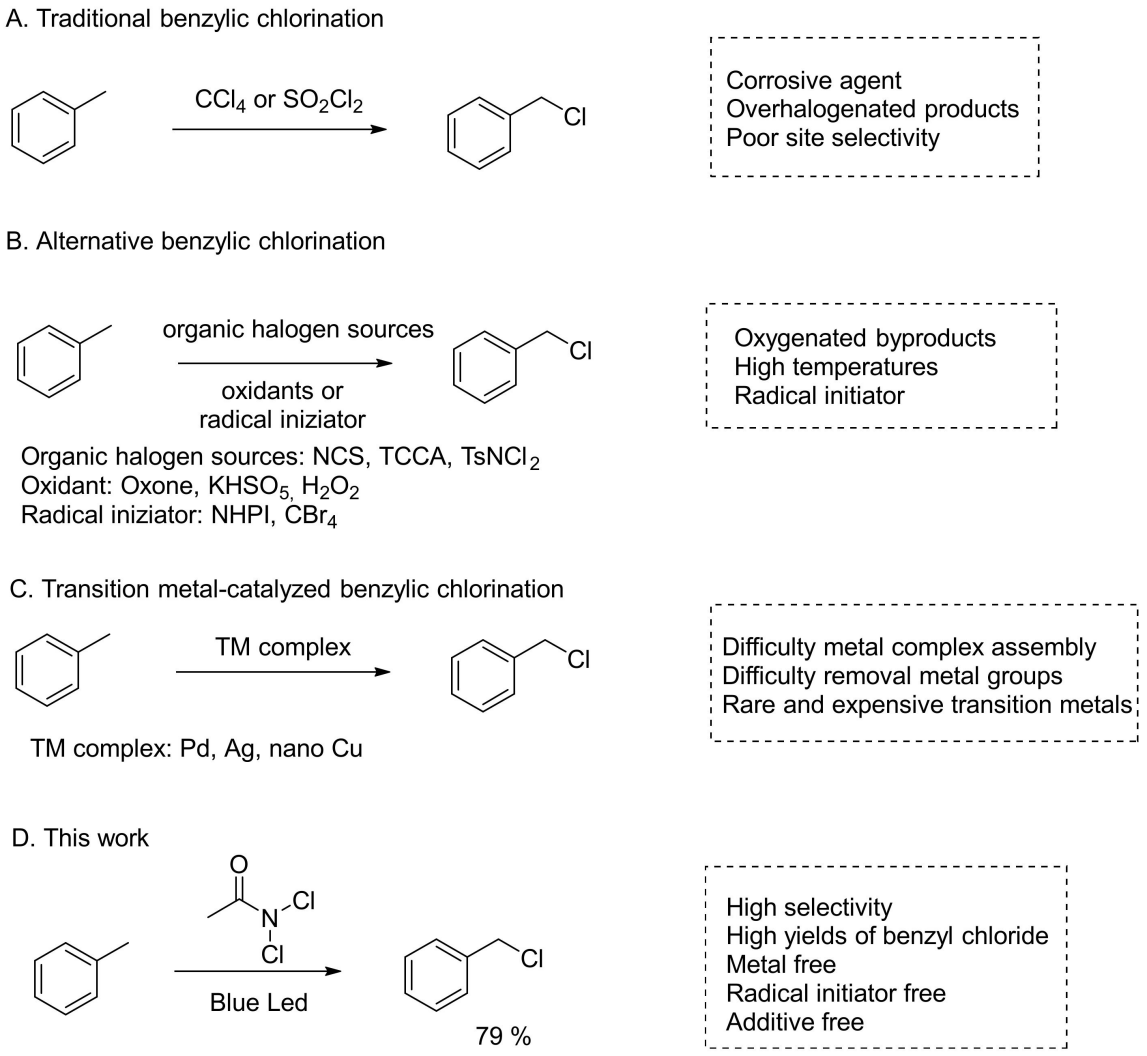
Strategies for the synthesis of benzyl chloride.

Other traditional synthetic strategies involve benzylic chlorination using explosive or corrosive chlorinating reagents such as CCl_4_ or SO_2_Cl_2_ (Scheme 1, pathway A) [13,14,15]. Moreover, the reagent concentrations and reaction conditions must be carefully controlled to minimize the formation of overhalogenated products and isomers [13].

For these reasons, academic and industrial researchers continue to study and to develop alternative approaches that enable the α-chlorination of toluene and alkyl arenes to achieve high conversion and yields of benzyl chloride, using mild and easy-to-handle reagents.

A more controlled synthesis method for halogenated chemicals, under relatively mild reaction conditions, is provided by using organic halogen sources (e.g., *N*-chlorosuccinimide (NCS), trichloroisocyanuric acid (TCCA) and dichloramine-T (TsNCl_2_)) (Scheme 1, pathway B) [16,17,18,19,20,21,22,23]. Thirumamagal et al. have reported the reaction of xylene with *N*-chlorosuccinimide (NCS) as a reagent and montmorillonite clay K-10 as a solid support in 1,2-dichloroethane [19]. The reaction was heated for 18 h at 80 °C, giving the desired products with a yield range of 63 to 67% [19]. In 2010, Bucos et al. proposed the microwave-assisted chlorination of alkyl aromatic hydrocarbons with *N*-chlorosuccinimide (NCS) in the presence of ionic species as additives [20]. This reaction has been studied only on *p*-xylene, but it was not possible to obtain 1-(chloromethyl)-4-methylbenzene as the exclusive product. In fact, the desired product was obtained with yields ranging from 3% to 50%.

Other methods for benzyl chlorination involve the combination of chloride anion and stoichiometric amounts of oxidants, such as peroxides, Oxone, KHSO_5_ and H_2_O_2_, under acidic or basic reaction conditions, which can avoid the use of toxic chlorinating reagents and afford low concentrations of chlorine radicals [24,25,26,27]. Unfortunately, these reactions often require high temperatures to release chlorine radicals (Cl•) to attack C–H bonds. Furthermore, it is often necessary to work with unfavorable stoichiometric ratios, and they often give oxygenated by-products (Scheme 1, pathway B). Li et al. have reported the chlorination reaction of toluenes using *N*-chlorosuccinimide (NCS) and *N*-hydroxyphthalimide (NHPI) as a catalyst and 2,3-dicyano-5,6-dichloro-benzoquinone (DDQ) as an external radical initiator by heating at 80 °C [22].

A current trend in organic chemistry is the improvement of new methodologies induced by visible light [28]. In this way, it is possible to develop sustainable and efficient synthetic methodologies. In fact, photosynthesis is the transformation of light into chemical energy and can be efficiently employed to promote chemical reactions. Photochemistry has long been a well-known part of traditional synthetic organic chemistry, but it has mainly been based on the direct excitation of organic compounds by UV light, which has significantly limited its scope. The use of visible light represents a very important goal for the organic chemist. In fact, visible light activates substrates without leaving by-products in the reaction mixture, with the significant simplification of the processing; for this reason, it can be considered a clean reagent. Furthermore, the use of visible light, instead of thermal energy, to carry out chemical processes leads to significant energy savings.

In this context, alternative strategies for site-selective chlorination at a specific C–H bond in an organic compound include transition metal-catalyzed C–H bond chlorination (Scheme 1, pathway C) [29,30]. Although transition metal-catalyzed direct C–H bond formation at C–Cl is an alternative and versatile strategy, it is more time-consuming due to the difficulties related to the complex synthesis and removal of the catalysts [31]. In particular, most of the reported transition metal-catalyzed C–H bond halogenation reactions have been achieved using rare and expensive transition metals, such as palladium and silver [32,33,34,35,36]. Stahl and co-workers reported copper-catalyzed benzyl chlorination with *N*-fluorobenzenesulfonimide as an oxidant [37]. This procedure shows benzyl site selectivity but requires a complex reaction system and an expensive ligand. Combe et al. proposed the chlorination of toluene using trichloroisocyanuric acid as a chlorine source, *N*-hydroxyphthalimide (NHPI) as a radical initiator and catalytic amounts of CBr_4_ and Cu(OAc)_2_ as catalysts, obtaining a benzyl chloride yield of 58% [17]. Whiting and co-workers reported the α-H-selective chlorination of alkylarenes using nano Ag/AgCl in aqueous NaCl/HCl under sunlight or visible light irradiation, obtaining benzyl chloride in a 38% yield [36]. Subsequently, the same group performed a selective photochlorination study using a nano-Cu@CuCl catalyst and various inorganic salts, under acidic conditions, obtaining benzyl chloride in yields ranging from 46 to 80% [38]. These methodologies, although interesting, do not allow one to obtain benzyl chloride with high yields; furthermore, it is necessary to prepare the catalyst with long procedures, sometimes even with difficult reaction conditions.

It is well known that C–H chlorination reactions have been widely employed to provide valuable building blocks in organic chemistry. Nevertheless, it is crucial to develop alternative and sustainable selective chlorination reactions of C–H bonds under visible-light irradiation.

Encouraged by our previous research results [21], we examined the use of inexpensive *N,N*-dichloroacetamide to induce the chlorination of alkylaromatic hydrocarbons via a photochemistry process. *N,N*-dichloroacetamide is a cheap and stable alternative to commercially available chlorinating reagents (Scheme 1, pathway D); the synthesis of *N,N*-dichloramide is simple, and, once prepared, it is easy to store, handle and use [39,40]. Furthermore, our work is metal-free and proceeds without the use of any additives or radical initiators. Moreover, the use of visible light allows for a sustainable and accessible alternative to traditional approaches and enables the introduction of new methods of reactivity in organic synthesis.

## 2. Results

### 2.1. Optimization of Reaction Conditions

We initially examined the chlorination of alkylaromatic hydrocarbons using toluene with *N,N*-dichloroacetamide (**2**) under different conditions (Table 1).

We began our investigation, as a model substrate, by using 2 mmol of toluene (**1**), with 1 mmol of *N,N*-dichloroacetamide (**2**), in 1 mL of dichloromethane as a solvent and irradiated by a blue LED (λ_max_ = 455 nm) for 8 h. Benzyl chloride (**3a**) was obtained in a 64% yield relative to the amount of the chlorine source. Increasing the amount of *N,N*-dichloroacetamide (**2**) to 1.2 mmol also increased the yield of benzyl chloride (**3a**) to 70% (Table 1, entry 2). An even better result was obtained by working with 1.3 mmol of *N,N*-dichloroacetamide (**2**); in this manner, it was possible to obtain benzyl chloride (**3a**) with a yield of 79% (Table 1, entry 3). Further increasing the amount of *N,N*-dichloroacetamide (**2**) to 2 mmol instead led to a slight decrease in the yield of the desired product (**3a**) to 70%, indicating that it was not necessary to work with such a high stoichiometric ratio (Table 1, entry 4). After having optimized the stoichiometric ratio between the reagents, the reaction was studied by varying the irradiation time. By irradiating the reaction mixture for 30 min, benzyl chloride (**3a**) was obtained in a 36% yield (Table 1, entry 5). By increasing the irradiation time to 1 h, benzyl chloride (**3a**) could be obtained in a 44% yield (Table 1, entry 6). Similar results were obtained by increasing the irradiation time to 2 h and 3 h, where benzyl chloride was obtained in 47% and 57% yields, respectively (Table 1, entries 7, 8). Encouraged by these results, the reaction was tested for 5 h, obtaining the corresponding chlorinated compound (**3a**) in a 65% yield. A further attempt by irradiating the reaction for up to 12 h did not lead to significant variations in yield (Table 1, entry 10). To improve the yield, some solvents were tested, but we were unable to obtain the desired product. In detail, cyclopentyl methyl ether (Table 1, entry 11), acetonitrile (Table 1, entry 12), 2-methyltetrahydrofuran (Table 1, entry 14), tetrahydrofuran (Table 1, entry 15) and 1,2-dichloroethane (Table 1, entry 16) were employed, but no product was obtained. Using ethyl acetate as a solvent, it was possible to obtain only trace amounts of benzyl chloride (**3a**) (Table 1, entry 13). Notably, the reaction did not proceed at all in the absence of light irradiation (Table 1, entry 17). The reaction was performed under green LED irradiation (Table 1, entry 18) and no product was obtained. This finding confirms the blue LED light activation process. The reaction was carried out in the air, but lower yields than under argon were obtained.

Finally, the reaction was tested by changing the chlorine source. Using *N,N*-dichloroformamide (**4**) and *N,N*-dichlorobenzamide (**5**), benzyl chloride was obtained with 47% and 60% yields, respectively.

**Scheme 2:**
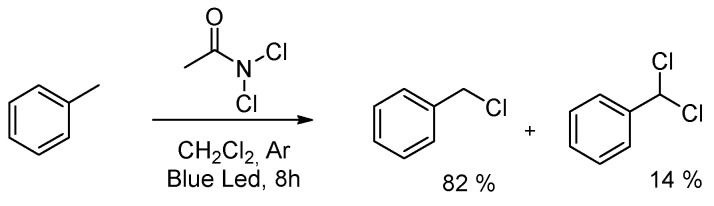
Evaluation of selectivity of toluene in benzyl chloride and benzal chloride.

We also studied the selectivity of toluene, evaluating the possible by-products that could be formed (Scheme 2). The method that was chosen to evaluate the selectivity was ^1^H NMR. With our procedure, it was possible to obtain selectivity of 96% of toluene. To our knowledge, this is one of the highest reported in the literature. Furthermore, it was possible to observe the selectivity in benzyl chloride of 82% and in benzal chloride of only 14%. No chlorination by-products of the benzene ring were observed.

### 2.2. Scale-Up of Our Methodology

We then evaluated the scale-up of our methodology. The reaction of 2.0 g of toluene with 1.8 g of *N,N*-dichloroacetamide in 10 mL of dichloromethane under blue LED irradiation for 8 h was tested. In this condition, benzyl chloride was obtained in a 74% yield. Both the yield and purity remained similar to the small-scale reaction. This result represents an important advantage of this approach, because one of the main disadvantages of many light-induced reactions is the need for the use of chromophores, which are such light absorbers, and this leads to scalability issues.

### 2.3. Scope of the Reaction

**Scheme 3:**
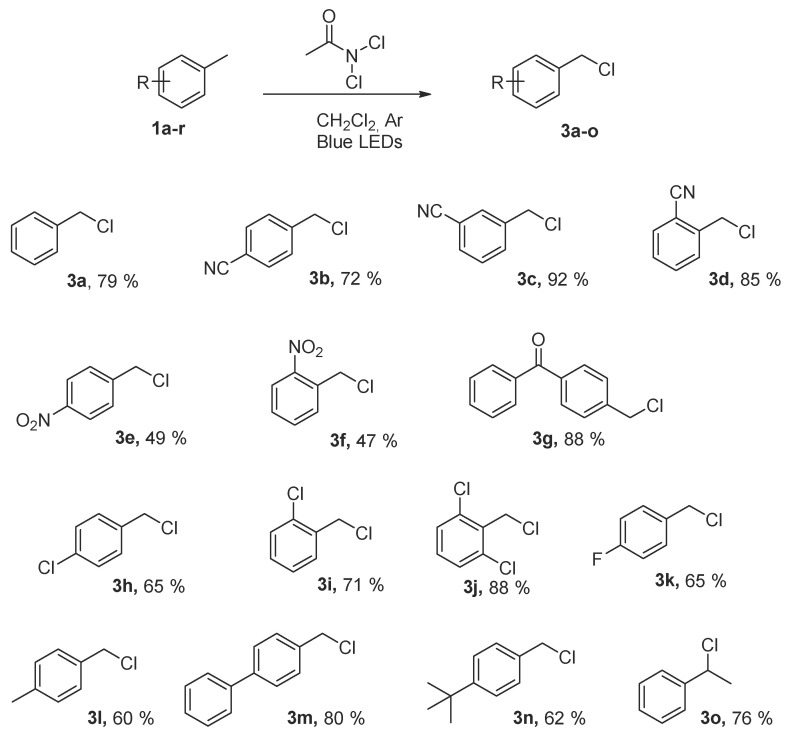
Substrate scope for the α-H chlorination of toluenes. Reaction conditions: toluenes (2.0 mmol), *N,N*-dichloroacetamide (**2**) (1.3 mmol), in DCM (1 mL), irradiated with blue LEDs for 8 h under Ar. Yields are for isolated products. Yields are relative to the amount of the chlorine source.

With the optimized reaction conditions in hand, the scope of the reaction was explored for variously substituted toluenes, affording a wide range of α-H-chlorinated alkylarenes (Scheme 3). In general, the corresponding α-H-chlorinated alkylarenes were obtained in satisfactory yields (Scheme 3, **3a**–**3o**). Different functional groups on the aromatic ring, both electron-donating and electron-withdrawing, were tested. Neither the electronic properties nor the steric effects of the substituents on the rings of alkylarenes were found to have effects on the reaction yields. Strong electron-withdrawing substituents were tested and showed very good results (Scheme 3, **3b**, **3c**, **3d**, **3e**, **3f**). CN groups in the ortho, meta and para positions were tested, giving the corresponding α-H-chlorinated alkylarene in very good yields (Scheme 3, **3b**, **3c**, **3d**). NO_2_ in the para and ortho positions also showed good results (Scheme 3, **3e**, **3f**). Moderate electron-withdrawing substituents in para, such as phenyl(p-tolyl)methanone, were employed, affording the corresponding (4-(chloromethyl)phenyl)(-phenyl)methanone (Scheme 3, **3g**) in an 88% yield. Toluenes with halide substituents, such as chlorine in the ortho, meta and para positions and fluorine in the para position, were tested to obtain the resultant α-H-chlorinated alkylarene (Scheme 3, **3h**, **3i**, **3j**, **3k**) in good yields. Substrates with moderate electron-donating substituents such as 4-methyl-1-1′-biphenyl and 1-(tert-butyl)-4-methylbenzene (Scheme 3, **3m**, **3n**) were tested, with satisfactory results. Para-xylene afforded the corresponding 1-(chloromethyl)-4-methylbenzene **3l** in a 60% yield by regioselectively chlorinating only at one position. The chlorination of ethylbenzene gave the corresponding (1-chloroethyl)benzene **3o** as the exclusive product in a 76% yield.

### 2.4. Proposed Mechanism

**Scheme 4:**
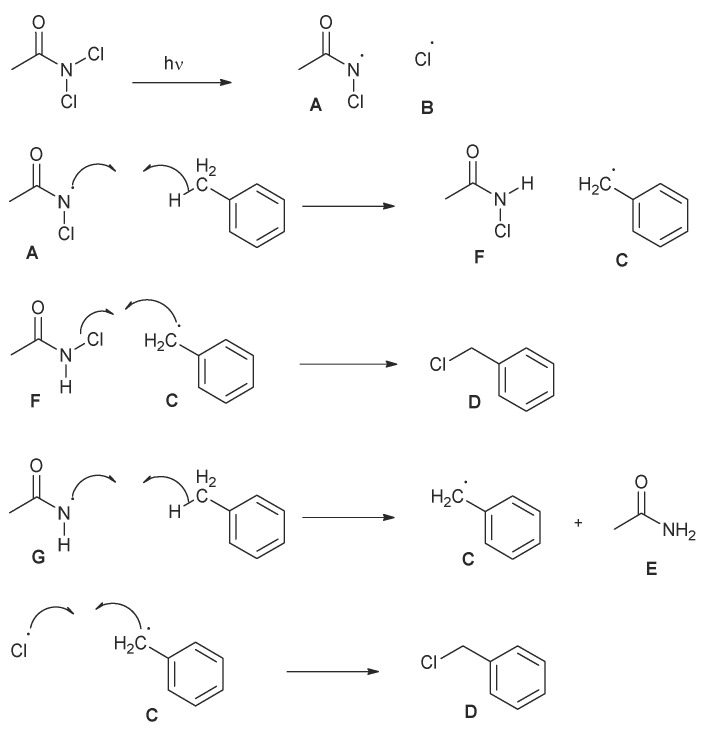
Plausible mechanism for the α-chlorination of toluene.

Based on previous studies [21,41,42], a description of a possible mechanism is reported in Scheme 4. A radical mechanism is supposed [42,43,44].

The reaction starts with the homolytic cleavage of the N–Cl bond of *N,N*-dichloroacetamide to generate radical chlorine atom **B** and nitrogen-centered radical **A** [45,46,47]. Then, the process propagates via the abstraction of toluene’s hydrogen by **A** to generate intermediate benzyl radical **C** and *N*-chloroacetamide **F**. Then, acyl radical **C** reacts with *N*-chloroacetamide **F** to generate benzyl chloride **D** and nitrogen-centered radical **F**. Subsequently, nitrogen-centered radical **F** reacts with toluene to give benzyl radical **C** and acetamide **E**. Finally, benzyl radical **C** is quenched by the radical chlorine atom **B** to generate benzyl chloride **D**.

## 3. Materials and Methods

### 3.1. General Information

All solvents and reagents were employed as purchased from commercial suppliers. All reactions were carried out in an argon atmosphere using standard methods. All solvents were dried by common techniques and distilled in an argon atmosphere. Short-column chromatography was performed with a 4 cm column diameter charged with 18 g of silica gel (pore size 60 Å, 32–63 nm particle size), and reactions were monitored by thin-layer chromatography (TLC) analysis, which was carried out with Merck Kieselgel 60 F254 plates and visualized using UV light at 254 nm. Irradiation with blue light was performed with the OSRAM Oslon SSL 80 LDCQ7P-1U3U (blue, λ_max_ = 455 nm, I_max_ = 1000 mA, 1.12 W). ^1^H NMR and ^13^C NMR spectra were recorded by a Bruker Avance III 400 spectrometer (400 MHz or 100 MHz, respectively) using CDCl_3_ solutions and TMS as an internal standard. Chemical shifts are reported in parts per million (ppm, d) relative to the internal tetramethylsilane standard (TMS, d 0.00). The peak patterns are denoted as follows: s, singlet; d, doublet; t, triplet; m, multiplet; q, quartet; dd, doublet of doublets; br, broad. The coupling constants, *J*, are indicated in Hertz (Hz). Melting points were recorded in open capillary tubes and were uncorrected.

### 3.2. General Procedure for N,N-Dichloroamides 2, 4, 5

In a round-bottom flask of 10 mL [39], we added 1 mmol amide and 2 mmol (0.21 g) of *tert*-Butyl hypochlorite [40] in 2 mL of diethyl ether at room temperature. The resulting suspension was stirred for 2 h. The reaction was monitored by TLC. After 2 h, the crude mixture was purified by short-column chromatography (diameter = 4 cm with 18 g of silica gel, Hexane/AcOEt).

### 3.3. General Procedure for Compounds ***3a**–**3o***

In a round-bottom flask of 10 mL, we added 2 mmol of alkylarenes and 1.3 mmol (0.166 g) of *N,N*-dichloroacetamide in 1 mL of dichloromethane under a dry argon atmosphere at room temperature. The resulting suspension was irradiated under a blue LED, under stirring, for 8 h. The reaction was monitored by TLC. After 8 h, the crude mixture was purified by short-column chromatography (diameter = 4 cm with 18 g of silica gel, Hexane/AcOEt). Detailed experimental steps and NMR results can be viewed in the Supplementary Materials.

## 4. Conclusions

In conclusion, the visible-light-mediated synthesis of α-H chloroalkylaromatic hydrocarbons has been reported. The conditions are mild and the stoichiometric ratio of the reagents is ideal. The methodology has shown to have good versatility and applicability for substrates with various functional groups, providing an alternative approach to the visible-light-mediated synthesis of α-H chloroalkylaromatic hydrocarbons. The use of visible light allows for a sustainable and accessible alternative to traditional approaches. Additionally, the methodology is metal-free and proceeds without the use of any additives and radical initiators. Moreover, the products can be obtained in very good yields.

## Figures and Tables

**Table 1:** Screening of reaction conditions.

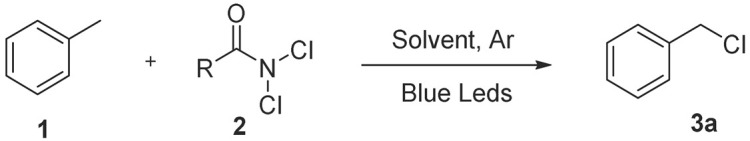					
Entry ^1^	Toluene (mmol)	Cl Source (mmol)	Solvent	Time (h)	Yield ^2,5^ (%)
1	2	(**2**) 1	CH_2_Cl_2_	8	64%
2	2	(**2**) 1.2	CH_2_Cl_2_	8	70%
3	2	(**2**) 1.3	CH_2_Cl_2_	8	79%
4	2	(**2**) 2	CH_2_Cl_2_	8	70%
5	2	(**2**) 1.3	CH_2_Cl_2_	0.5	36%
6	2	(**2**) 1.3	CH_2_Cl_2_	1	44%
7	2	(**2**) 1.3	CH_2_Cl_2_	2	47%
8	2	(**2**) 1.3	CH_2_Cl_2_	3	57%
9	2	(**2**) 1.3	CH_2_Cl_2_	5	65%
10	2	(**2**) 1.3	CH_2_Cl_2_	12	66%
11	2	(**2**) 1.3	CPME	8	-
12	2	(**2**) 1.3	CH_3_CN	8	-
13	2	(**2**) 1.3	AcOEt	8	trace
14	2	(**2**) 1.3	2-MeTHF	8	-
15	2	(**2**) 1.3	THF	8	-
16	2	(**2**) 1.3	DCE	8	-
17 ^3^	2	(**2**) 1.3	CH_2_Cl_2_	8	-
18 ^4^	2	(**2**) 1.3	CH_2_Cl_2_	8	-
19	2	(**4**) 1.3	CH_2_Cl_2_	8	47%
20	2	(**5**) 1.3	CH_2_Cl_2_	8	60%
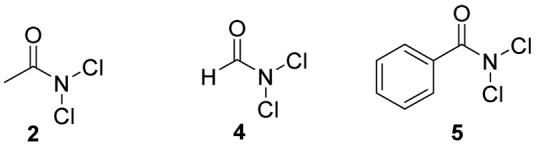					

^1^ Reaction conditions: toluene (2.0 mmol), Cl source (1.3 mmol), in solvent (1 mL), irradiated with blue LEDs for 8 h under Ar. ^2^ Yields are for isolated products. ^3^ Reaction performed without light. ^4^ The reaction mixture was irradiated for 8 h with a green LED (535 nm). ^5^ Yields are relative to the amount of the chlorine source.

## Data Availability

The data presented in this study are available in the Supplementary Materials.

## Supplementary Materials

The following supporting information can be downloaded at: https://www.mdpi.com/article/10.3390/molecules30020312/s1, Experimental Section; General Information; General Procedure for evaluation of conversion of toluene; General Procedure to *N,N*-dichloroamides 2, 4, 5; General Procedure to compounds **3a**–**3o**; Compound characterizations **3a**–**3o**; Experimental Set-up; References [9,17,27,39,40,48,49,50,51,52,53]; NMR Spectra.
