# Supplementary material for: Metal-Free Synthesis of α-H Chlorine Alkylaromatic Hydrocarbons Driven by Visible Light
Source: Molecules. 2025 Jan 14;30(2):312. doi: 10.3390/molecules30020312 (PMC11768015; doi:10.3390/molecules30020312)

Supporting Information

Metal-free synthesis of α-H chlorine alkylaromatic hydrocarbons driven by visible light

Lidia De Luca [1], Luca Ledda [1], Andrea Porcheddu [2], Massimo Carraro [1], Luisa Pisano [1] and Silvia Gaspa [1]*

[1] Dipartimento di Scienze Chimiche, Fisiche, Matematiche e Naturali, Università degli Studi di Sassari, Via Vienna 2, 07100, Sassari, Italy; ldeluca@uniss.it (L.D.L.); lucaledda@gmail.com (L.L.), mcarraro@uniss.it (M.C.), luisa@uniss.it (L.P.) sgaspa@uniss.it (S.G.)
[2] Dipartimento di Scienze Chimiche e Geologiche, Università degli Studi di Cagliari, Cittadella Universi-taria, 09042 Monserrato, Italy; porcheddu@unica.it
* Correspondence: sgaspa@uniss.it

Table of contents

Experimental section……………………………………………………………………... S4

General information…………………………………………………………………… S4

General Procedure for evaluation of conversion of toluene……………………………… S4

General Procedure to *N,N*-dichloroamides 2, 4, 5………………………………………… S4

General Procedure to compounds 3a-3o…………………………………………………… S5

Compound characterizations 3a-3o……………………………………………………... S6

Experimental Set-up……………………………………………………………………… S10

Reference………………………………………………………………………………….. S11

NMR Spectra ^{1}H- ^{13}C……………………………………………………………….. S12

Experimental Section:

General Information

All solvents and reagents were employed as bought by commercial suppliers. All the reactions were carried out in an Argon atmosphere using standard methods. All solvents were dried by common techniques and distilled in an Argon atmosphere. Short-column chromatography was performed with 4 cm column diameter charged with 18 g of silica gel (pore size 60 Å, 32-63 nm particle size) and reactions were monitored by thin-layer chromatography (TLC) analysis was carried out with Merck Kieselgel 60 F254 plates and visualized using UV light at 254 nm. Irradiation with blue light was performed with OSRAM Oslon SSL 80 LDCQ7P-1U3U (blue, λ max = 455 nm, I max = 1000 mA, 1.12 W). ^{1}H NMR and ^{13}C NMR spectra were recorded by a Bruker Avance III 400 spectrometer (400 MHz or 100 MHz, respectively) using $CDCl_3$ solutions and TMS as an internal standard. Chemical shifts are reported in parts per million (ppm, d) relative to the internal tetramethylsilane standard (TMS, d 0.00). The peak patterns are denoted as follows: s, singlet; d, doublet; t, triplet; m, multiplet; q, quartet; dd, doublet of doublets; br, broad. The coupling constants, *J*, are indicated in Hertz (Hz). Melting points were recorded in open capillary tubes and were uncorrected.

General Procedure for evaluation of conversion of toluene:

In a round bottom flask of 10 mL were added 2 mmol (0.194 g) of toluene and 1.3 mmol (0.166 g) of *N,N*-dichloroacetamide in 1 mL of dichloromethane under dry Argon atmosphere at room temperature. The resulting suspension was irradiated under blue LED, under stirring, for 8 h. The crude product was analyzed by ^{1}H-NMR and the conversion was calculated as follow:

$$^{1}H\ NMR\ Conversion = \frac{\frac{1}{2}\ Area\ of\ CH_2\ benzyl\ chloride}{\frac{1}{2}Area\ of\ CH_2\ benzyl\ chloride + \frac{1}{3}Area\ of\ CH_3\ toluene + Area\ of\ CH\ benzal\ chloride}$$

After 8 hours it was possible to observe 82:14:4 of benzyl chloride, benzal chloride and unreacted toluene.

General Procedure to *N,N*-dichloroamides 2, 4, 5:[1]

In a round bottom flask of 10 mL were added 1 mmol amide and 2 mmol (0.21 g) of *tert*-Butyl hypochlorite[2] in 2 mL of diethyl ether at room temperature. The resulting suspension was stirred for 2 h. The reaction was monitored by TLC. After 2 h, the crude mixture was purified by short column chromatography (diameter= 4 cm with 18 g of silica gel, Hexane/AcOEt).

N,N-dichloroacetamide (2): ^{1}H NMR (400 MHz, $CDCl_3$) δ: 2.40 (s, 3H).

General Procedure to compounds 3a-3o:

In a round bottom flask of 10 mL were added 2 mmol of alkylarenes and 1.3 mmol (0.166 g) of *N,N*-dichloroacetamide in 1 mL of dichloromethane under dry Argon atmosphere at room temperature. The resulting suspension was irradiated under blue LED, under stirring, for 8 h. The reaction was monitored by TLC. After 8 h, the crude mixture was purified by short column chromatography (diameter= 4 cm with 18 g of silica gel, Hexane/AcOEt).

Compound characterizations 3a-3o:

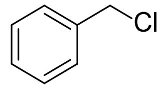

(Chloromethyl)benzene (3a):[3] Colorless oil; (79 % yield); R_f= 0.68 (hexane/ethyl acetate 4.8/0.2). ^{1}H NMR (400 MHz, $CDCl_3$) δ: 7.48 – 7.29 (m, 5H), 4.61 (s, 2H). ^{13}C NMR (100 MHz, $CDCl_3$) δ: 137.5, 128.7, 128.5, 128.4, 46.2.

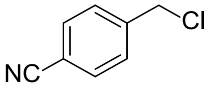

4-(chloromethyl)benzonitrile (3b):[3] Colorless solid; (72 % yield); m.p. 79 - 81 °C, R_f = 0.178 (hexane/ethyl acetate 4.8/0.2). ^{1}H NMR (400 MHz, $CDCl_3$) δ: 7.66 (d, J = 8.1 Hz, 2H), 7.50 (d, J = 8.1 Hz, 2H), 4.60 (s, 2H). ^{13}C NMR (100 MHz, $CDCl_3$) δ: 142.4, 132.5, 129.1, 118.3, 112.2, 44.9.

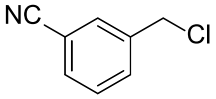

3-(chloromethyl)benzonitrile (3c):[4] White solid; (92 % yield); m.p. 72 - 74 °C, R_f= 0.28 (hexane/ethyl acetate 4.5/0.5). ^{1}H NMR (400 MHz, $CDCl_3$) δ: 7.69 (s, 1H), 7.64 – 7.61 (m, 2H), 7.49 (t, J = 7.8 Hz, 1H), 4.59 (s, 2H). ^{13}C NMR (100 MHz, $CDCl_3$) δ: 138.9, 132.8, 132.0, 132.0, 129.6, 118.2, 113.0, 44.6.

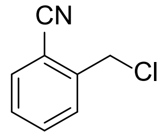

2-(chloromethyl)benzonitrile (3d):[5] Colorless oil; (85 % yield); R_f= 0.3 (hexane/ethyl acetate 4.5/0.5). ^{1}H NMR (400 MHz, $CDCl_3$) δ: 7.68 (d, J = 7.7 Hz, 1H), 7.65 – 7.57 (m, 2H), 7.44 (t, J = 8.0 Hz, 1H), 4.76 (s, 2H). ^{13}C NMR (100 MHz, $CDCl_3$) δ: 140.7, 133.2, 133.0, 130.0, 129.0, 116.7, 112.3, 43.1.

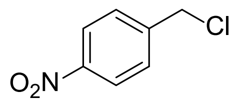

1-(chloromethyl)-4-nitrobenzene (3e):[6] White solid; (49 % yield); m.p. 70 - 72 °C, R_f = 0.419 (hexane/ethyl acetate 4.8/0.2). ^{1}H NMR (400 MHz, $CDCl_3$) δ: 8.22 (d, J = 8.6 Hz, 2H), 7.57 (d, J = 8.5 Hz, 2H), 4.65 (s, 2H). ^{13}C NMR (100 MHz, $CDCl_3$) δ: 147.8, 144.3, 129.3, 123.9, 44.5.

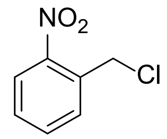

1-(chloromethyl)-2-nitrobenzene (3f):[7] Colorless oil; (47 % yield); R_f = 0.3 (hexane/ethyl acetate 4.8/0.2). ^{1}H NMR (400 MHz, $CDCl_3$) δ: 8.06 (d, J = 8.2 Hz, 1H), 7.71 – 7.64 (m, 2H), 7.51 (t, J = 7.6 Hz, 1H), 4.98 (s, 2H). ^{13}C NMR (100 MHz, $CDCl_3$) δ: 148.0, 133.7, 132.4, 131.6, 129.4, 125.2, 42.8.

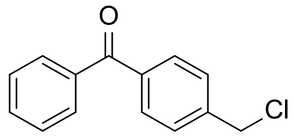

(4-(chloromethyl)phenyl)(phenyl)methanone (3g):[8] Yellow oil; (88 % yield); R_f= 0.407 (hexane/ethyl acetate 4.5/0.5). ^{1}H NMR (400 MHz, $CDCl_3$) δ: 7.81 – 7.79 (m, 4H), 7.62 – 7.58 (m, 1H), 7.53 – 7.45 (m, 4H), 4.65 (s, 2H). ^{13}C NMR (100 MHz, $CDCl_3$) δ: 196.0, 141.7, 137.5, 137.4, 132.5, 130.5, 130.0, 128.4, 128.3, 45.4.

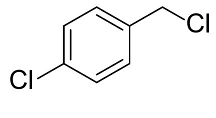

1-chloro-4-(chloromethyl)benzene (3h):[3] Colorless oil; (65 % yield); R_f= 0.75 (hexane/ethyl acetate 4.8/0.2). ^{1}H NMR (400 MHz, $CDCl_3$) δ: 7.35 - 7-31 (m, 4H), 4.55 (s, 2H). ^{13}C NMR (100 MHz, $CDCl_3$) δ: 135.9, 134.3, 129.9, 128.9, 45.3.

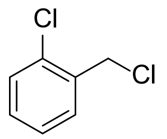

1-chloro-2-(chloromethyl)benzene (3i):[3] Colorless oil; (71 % yield); R_f= 0.67 (hexane/ethyl acetate 4.8/0.2). ^{1}H NMR (400 MHz, $CDCl_3$) δ: 7.53 – 7.51 (m, 1H), 7.47 – 7.45 (m, 1H), 7.34 – 7.32 (m, 2H), 4.77 (s, 2H). ^{13}C NMR (100 MHz, $CDCl_3$) δ: 134.9, 133.9, 130.7 129.8, 129.7, 127.1, 43.5.

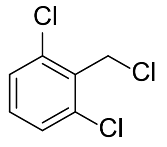

1,3-dichloro-2-(chloromethyl)benzene (3j):[9] Colorless oil; (88 % yield); R_f= 0.586 (hexane/ethyl acetate 4.5/0.5). ^{1}H NMR (400 MHz, $CDCl_3$) δ: 7.34 (d, J = 8.0 Hz, 2H), 7.21 (t, J = 7.6 Hz, 1H), 4.88 (s, 2H). ^{13}C NMR (100 MHz, $CDCl_3$) δ: 136.1, 133.4, 130.2, 128.5, 40.7.

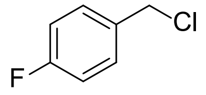

1-(chloromethyl)-4-fluorobenzene (3k):[10] Colorless oil; (65 % yield); R_f= 0.48 (hexane/ethyl acetate 4.8/0.2). ^{1}H NMR (400 MHz, $CDCl_3$) δ: 7.37 (dd, J = 8.5, 5.3 Hz, 2H), 7.05 (t, J = 8.6 Hz, 2H), 4.57 (s, 2H). ^{13}C NMR (100 MHz, $CDCl_3$) δ: 162.6 (d, J = 246 Hz), 133.4 (d, J = 3 Hz), 130.4 (d, J = 8 Hz), 115.7 (d, J = 22 Hz), 45.5.

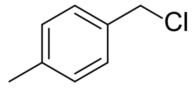

1-(chloromethyl)-4-methylbenzene (3l):[3] Colorless oil; (60 % yield); R_f= 0.66 (hexane/ethyl acetate 4.8/0.2). ^{1}H NMR (400 MHz, $CDCl_3$) δ: 7.29 (d, J = 7.9 Hz, 2H), 7.18 (d, J = 7.8 Hz, 2H), 4.58 (s, 2H), 2.37 (s, 3H). ^{13}C NMR (100 MHz, $CDCl_3$) δ: 138.3, 134.5, 129.4, 128.5, 46.3, 21.2.

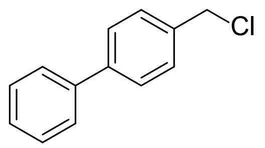

4-(chloromethyl)-1,1'-biphenyl (3m):[11] Colorless oil; (80 % yield); R_f = 0.5 (hexane/ethyl acetate 4.8/0.2). ^{1}H NMR (400 MHz, $CDCl_3$) δ: 7.60 (d, J = 7.7 Hz, 4H), 7.51 – 7.43 (m, 4H), 7.37 (t, J = 7.3 Hz, 1H), 4.65 (s, 2H). ^{13}C NMR (100 MHz, $CDCl_3$) δ: 141.4, 140.5, 136.4, 129.0, 128.8, 127.5, 127.5, 127.1, 46.0.

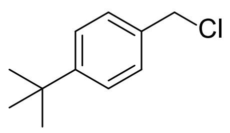

1-(*tert*-butyl)-4-(chloromethyl)benzene (3n):[6] Colorless oil; (62 % yield); R_f = 0.48 (hexane/ethyl acetate 4.9/0.1). ^{1}H NMR (400 MHz, $CDCl_3$) δ: 7.41 (d, J = 8.3 Hz, 2H), 7.35 (d, J = 8.2 Hz, 2H), 4.60 (s, 2H), 1.35 (s, 9H). ^{13}C NMR (100 MHz, $CDCl_3$) δ: 151.5, 134.5, 128.3, 125.7, 46.1, 34.6, 31.3.

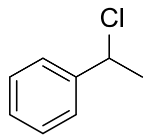

(1-chloroethyl)benzene (3o):[12] Colorless oil; (76 % yield); R_f = 0.6 (hexane/ethyl acetate 4.8/0.2). ^{1}H NMR (400 MHz, $CDCl_3$) δ: 7.44 – 7.30 (m, 5H), 5.10 (q, J = 6.8 Hz, 1H), 1.86 (d, J = 6.8 Hz, 3H). ^{13}C NMR (100 MHz, $CDCl_3$) δ: 142.8, 128.6, 128.2, 126.5, 58.8, 26.5.

Experimental Set-up:

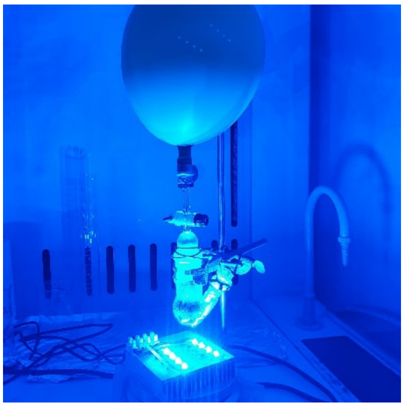

The light source used for photochemical experiments was Blue Led OSRAM Oslon;

Manufacturer: OSRAM Oslon; Model：Oslon SSL 80 LDCQ7P-1U3U λ max = 455 nm, I max = 1000 mA, 1.12 W

Material of the irradiation vessel borosilicate.

Distance from the light source to the irradiation vessel: 2.0 cm

References:

1. Zimmer, H.; Audrieth, L. F., Tertiary Butyl Hypochlorite as as N-Chlorinating Agent. *Journal of the American Chemical Society* **1954,** *76* (14), 3856-3857.
2. Gómez, J. E.; Guo, W.; Gaspa, S.; Kleij, A. W., Copper-Catalyzed Synthesis of γ-Amino Acids Featuring Quaternary Stereocenters. *Angewandte Chemie International Edition* **2017,** *56* (47), 15035-15038.
3. Combe, S. H.; Hosseini, A.; Parra, A.; Schreiner, P. R., Mild Aliphatic and Benzylic Hydrocarbon C–H Bond Chlorination Using Trichloroisocyanuric Acid. *The Journal of Organic Chemistry* **2017,** *82* (5), 2407-2413.
4. Guru, M. M.; Shima, T.; Hou, Z., Conversion of Dinitrogen to Nitriles at a Multinuclear Titanium Framework. *Angewandte Chemie International Edition* **2016,** *55* (40), 12316-12320.
5. Ozawa, J.; Kanai, M., Silver-Catalyzed C(sp3)–H Chlorination. *Organic Letters* **2017,** *19* (6), 1430-1433.
6. Huy, P. H.; Motsch, S.; Kappler, S. M., Formamides as Lewis Base Catalysts in SN Reactions—Efficient Transformation of Alcohols into Chlorides, Amines, and Ethers. *Angewandte Chemie International Edition* **2016,** *55* (34), 10145-10149.
7. Ding, R.; He, Y.; Wang, X.; Xu, J.; Chen, Y.; Feng, M.; Qi, C., Treatment of Alcohols with Tosyl Chloride Does Not always Lead to the Formation of Tosylates. *Molecules* **2011,** *16* (7), 5665.
8. Ogawa, D.; Hyodo, K.; Suetsugu, M.; Li, J.; Inoue, Y.; Fujisawa, M.; Iwasaki, M.; Takagi, K.; Nishihara, Y., Palladium-catalyzed and copper-mediated cross-coupling reaction of aryl- or alkenylboronic acids with acid chlorides under neutral conditions: efficient synthetic methods for diaryl ketones and chalcones at room temperature. *Tetrahedron* **2013,** *69* (12), 2565-2571.
9. Wang, Z.; Zhu, L.; Yin, F.; Su, Z.; Li, Z.; Li, C., Silver-Catalyzed Decarboxylative Chlorination of Aliphatic Carboxylic Acids. *Journal of the American Chemical Society* **2012,** *134* (9), 4258-4263.
10. HUY, P. H. A process for the preparation of halide derivatives. WO 2016/202894 Al, 2016.
11. Heijnen, D.; Tosi, F.; Vila, C.; Stuart, M. C. A.; Elsinga, P. H.; Szymanski, W.; Feringa, B. L., Oxygen Activated, Palladium Nanoparticle Catalyzed, Ultrafast Cross-Coupling of Organolithium Reagents. *Angewandte Chemie International Edition* **2017,** *56* (12), 3354-3359.
12. Zhao, M.; Lu, W., Visible Light-Induced Oxidative Chlorination of Alkyl sp3 C–H Bonds with NaCl/Oxone at Room Temperature. *Organic Letters* **2017,** *19* (17), 4560-4563.

NMR Spectra

(Chloromethyl)benzene (3a)

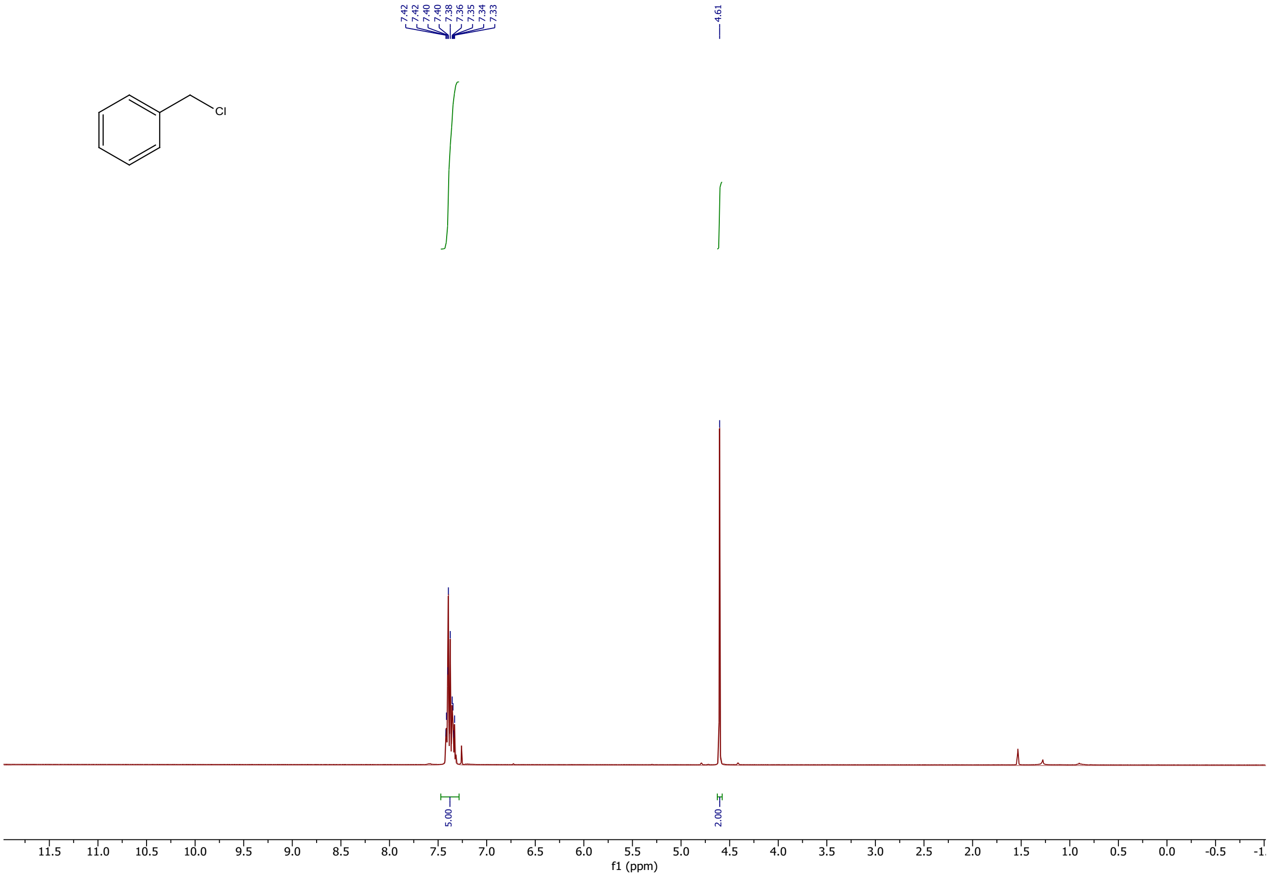

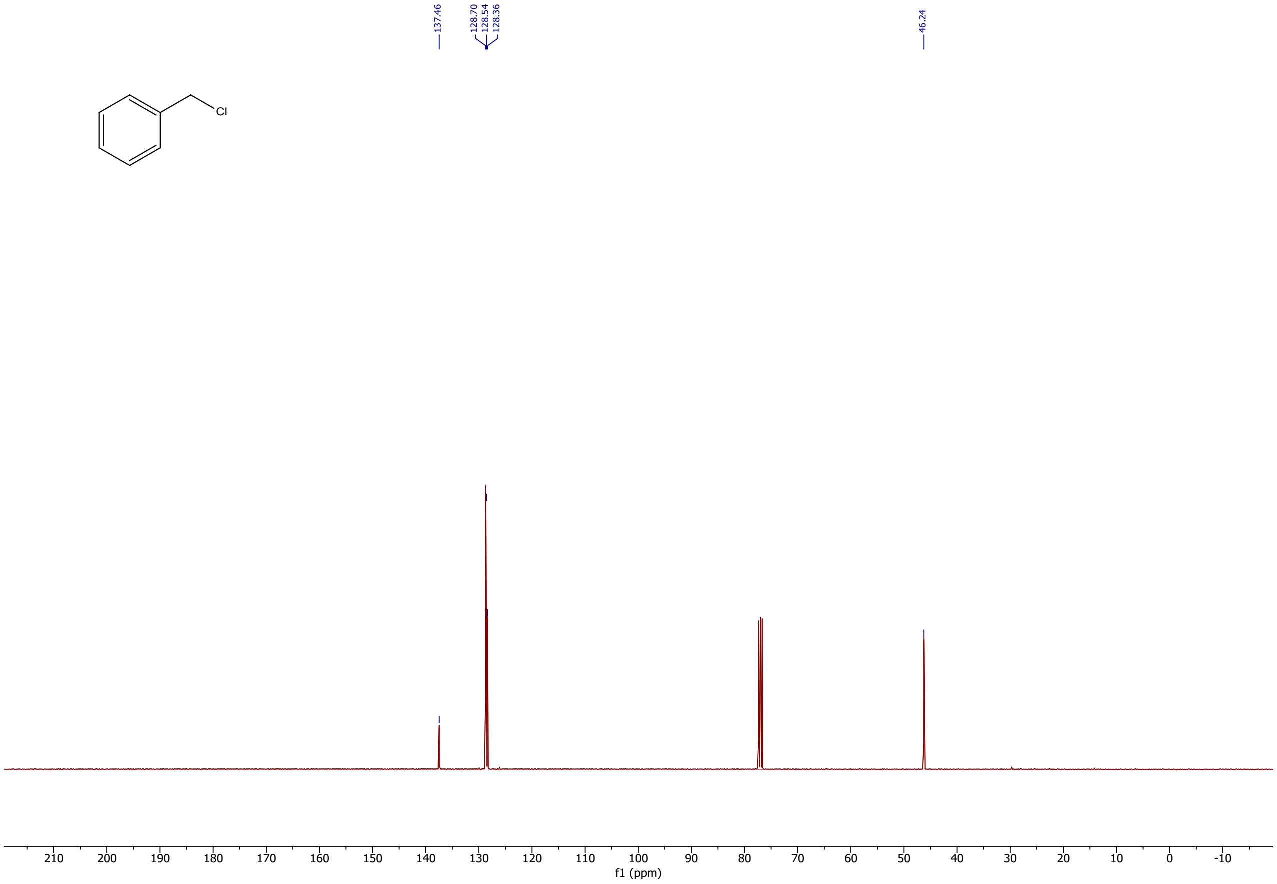

4-(chloromethyl)benzonitrile (3b)

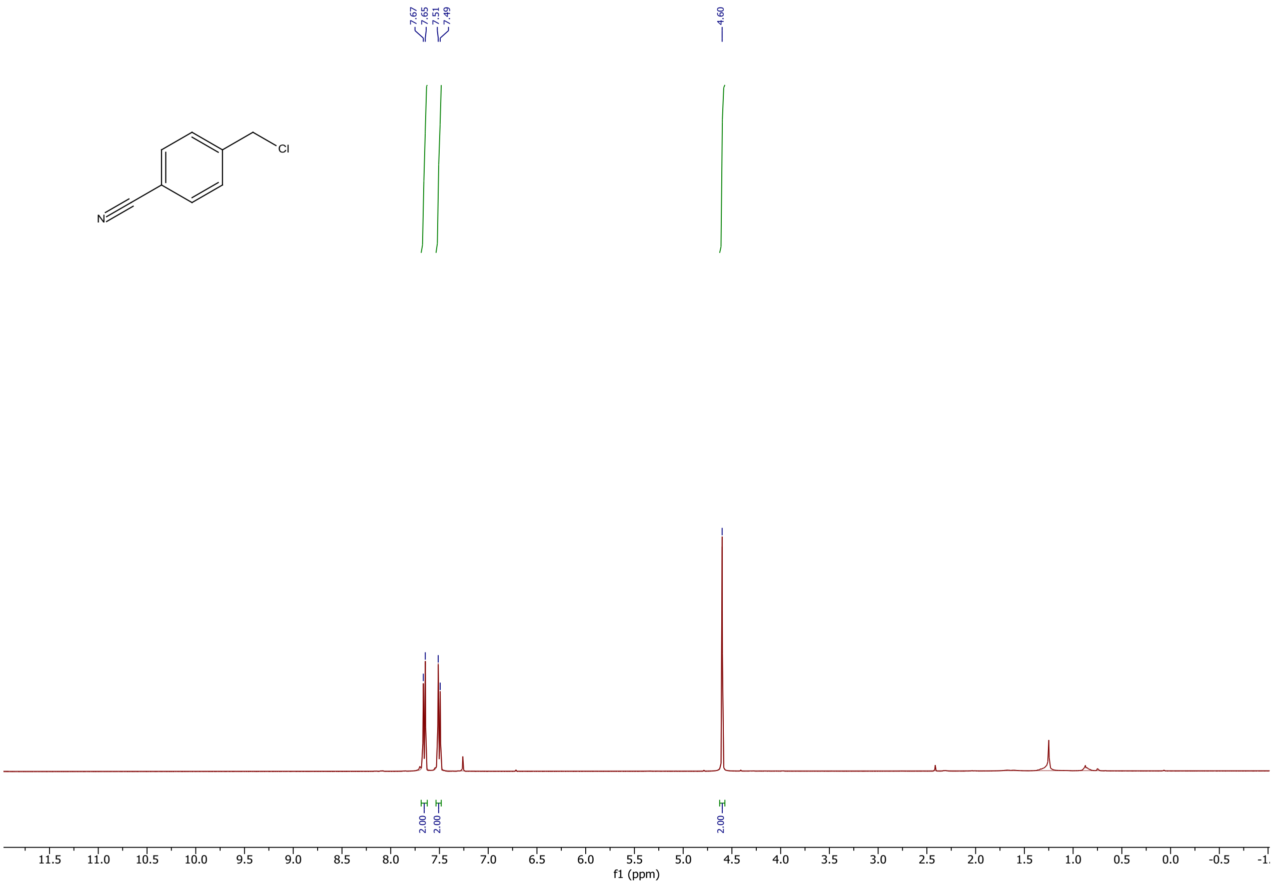

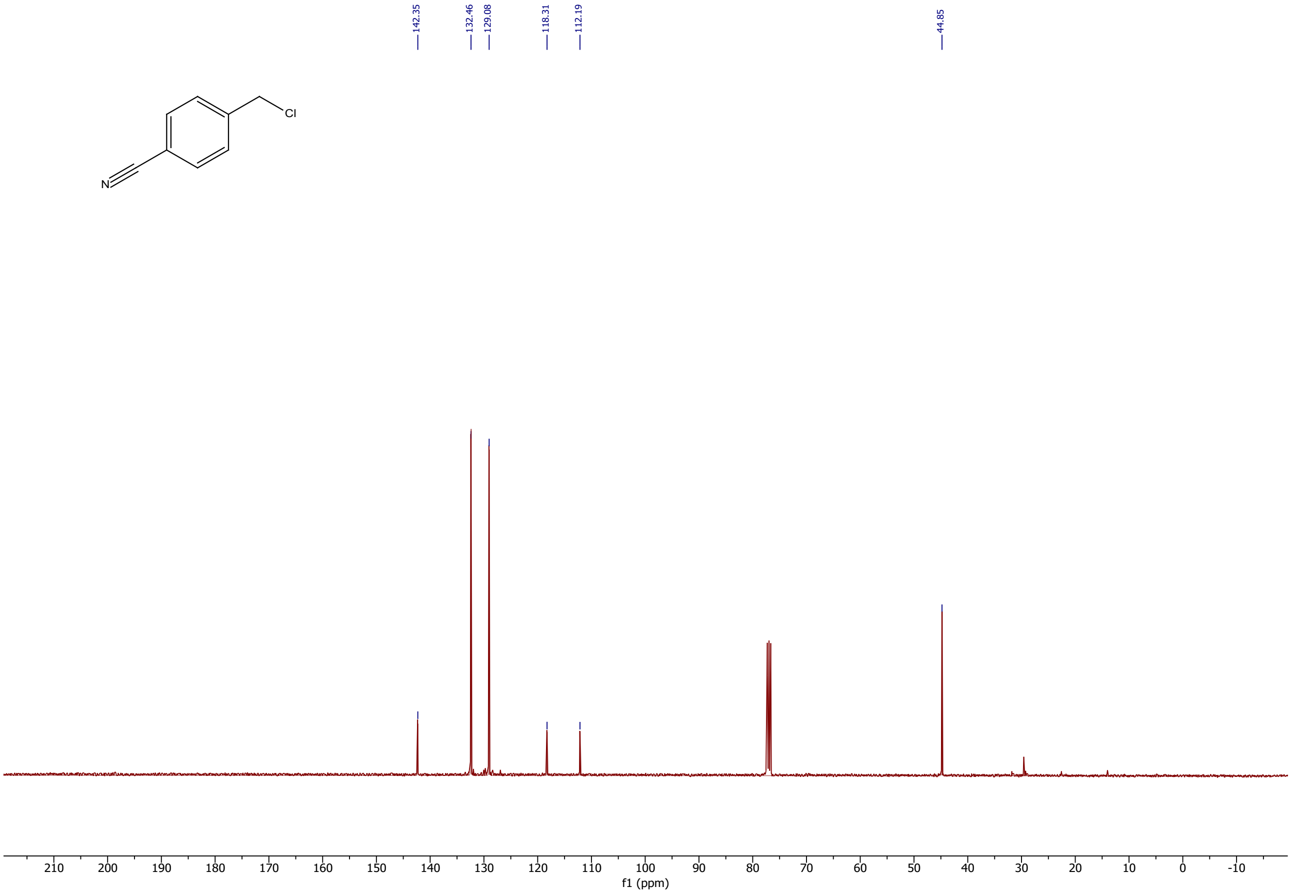

3-(chloromethyl)benzonitrile (3c)

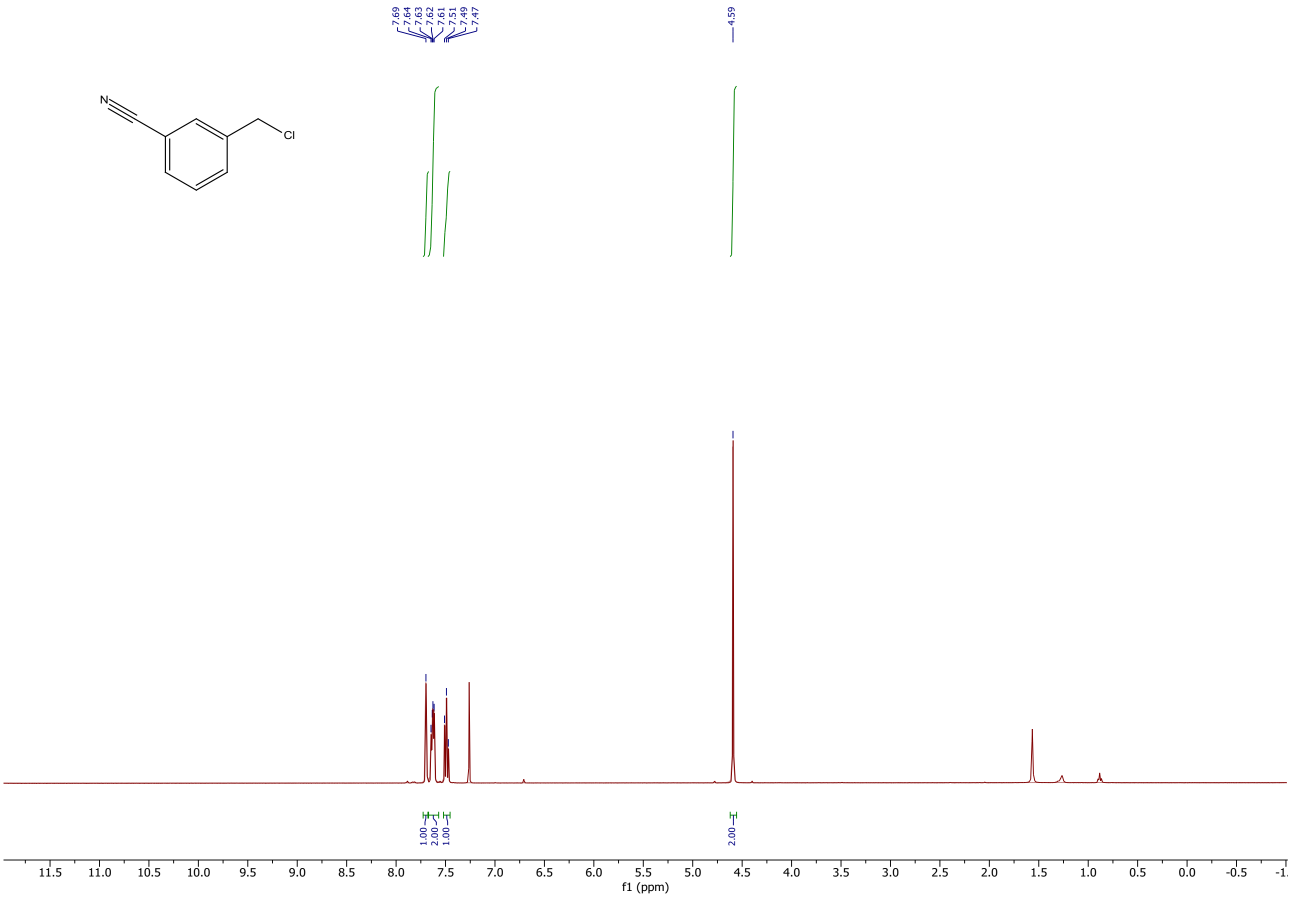

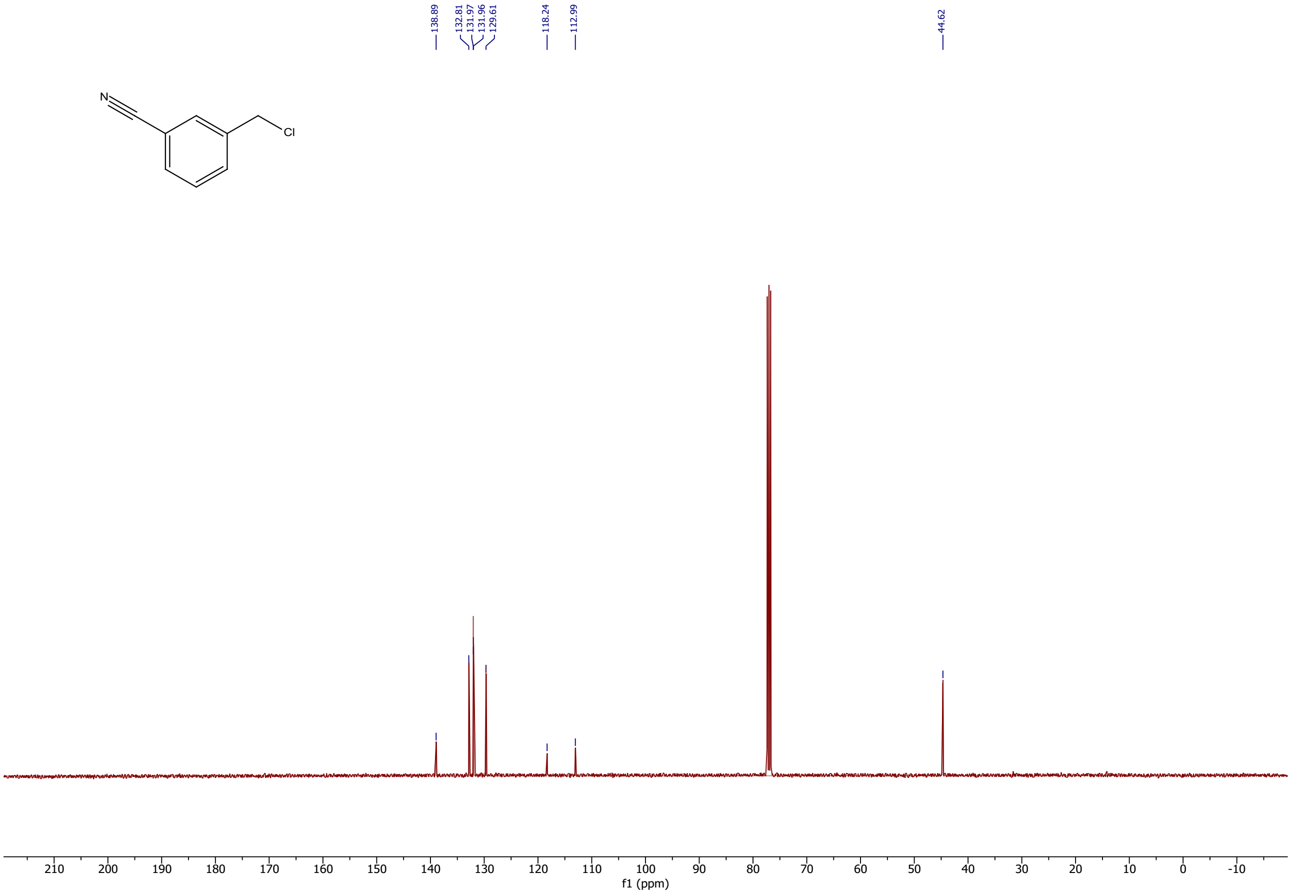

2-(chloromethyl)benzonitrile (3d)

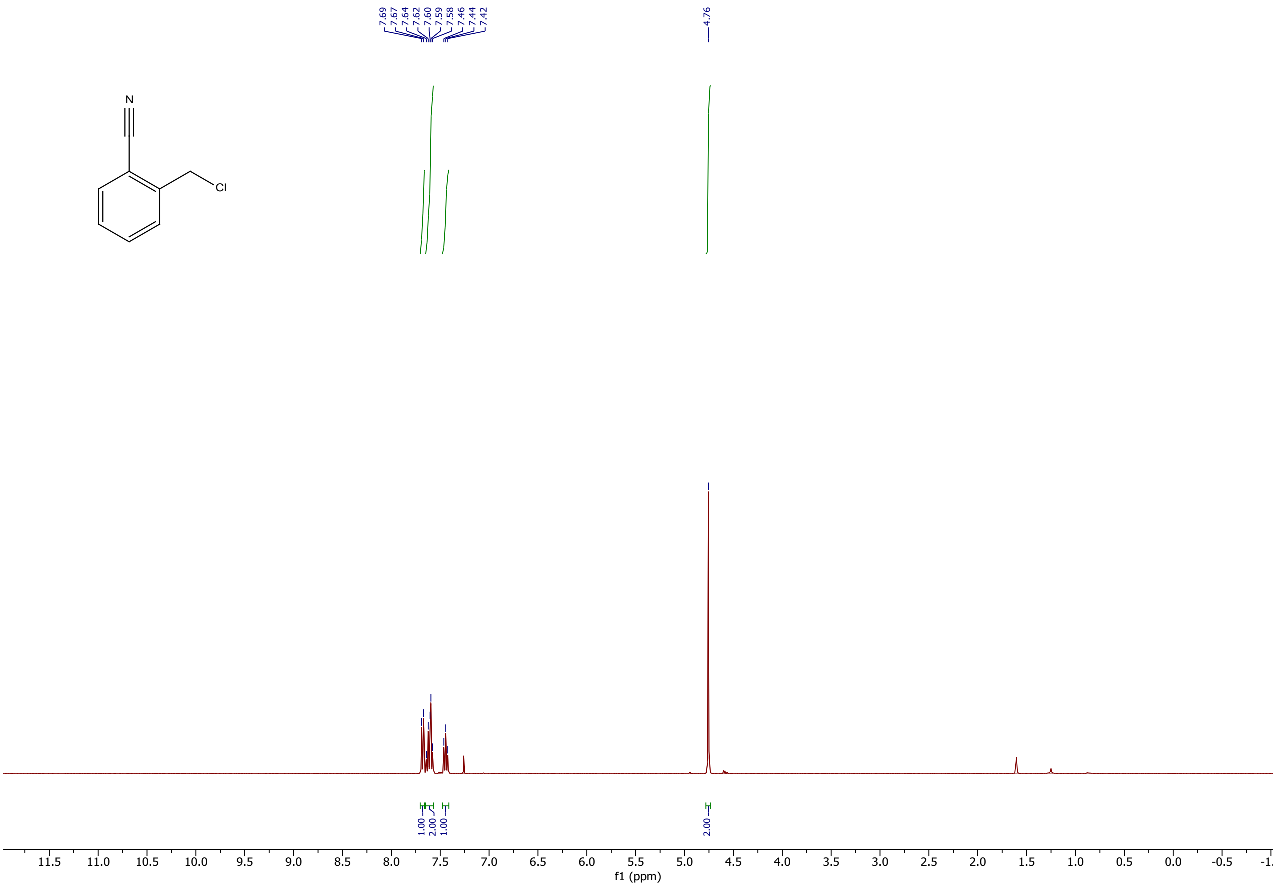

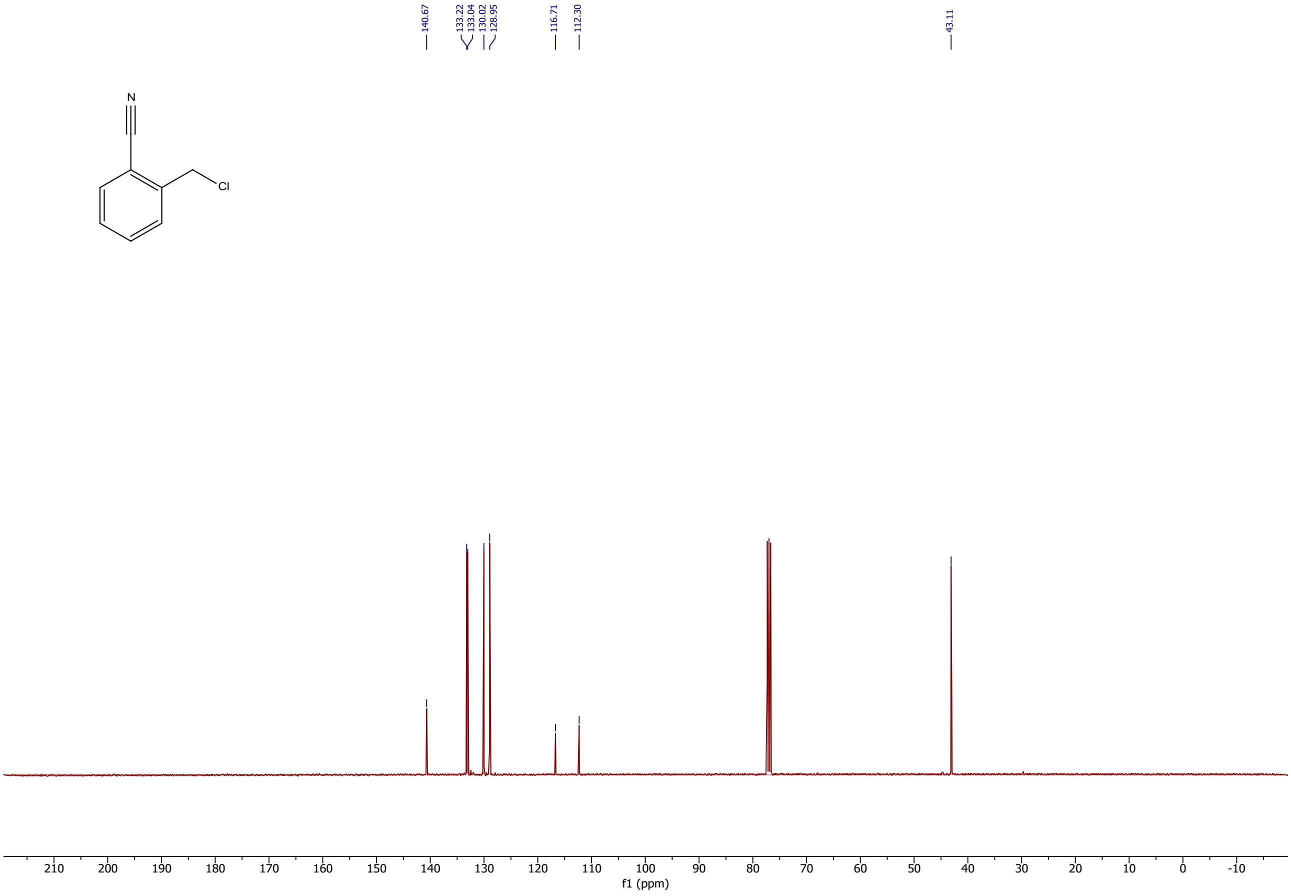

1-(chloromethyl)-4-nitrobenzene (3e)

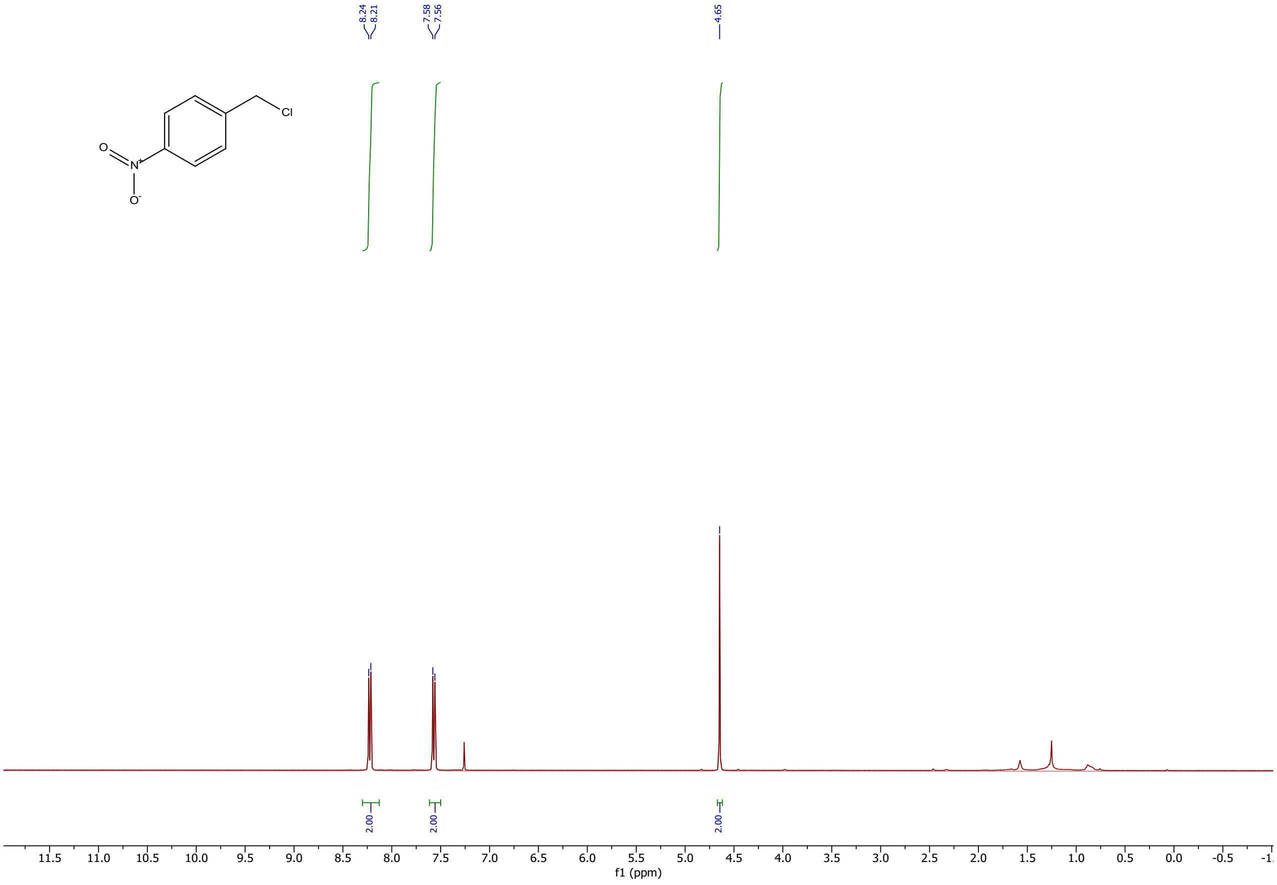

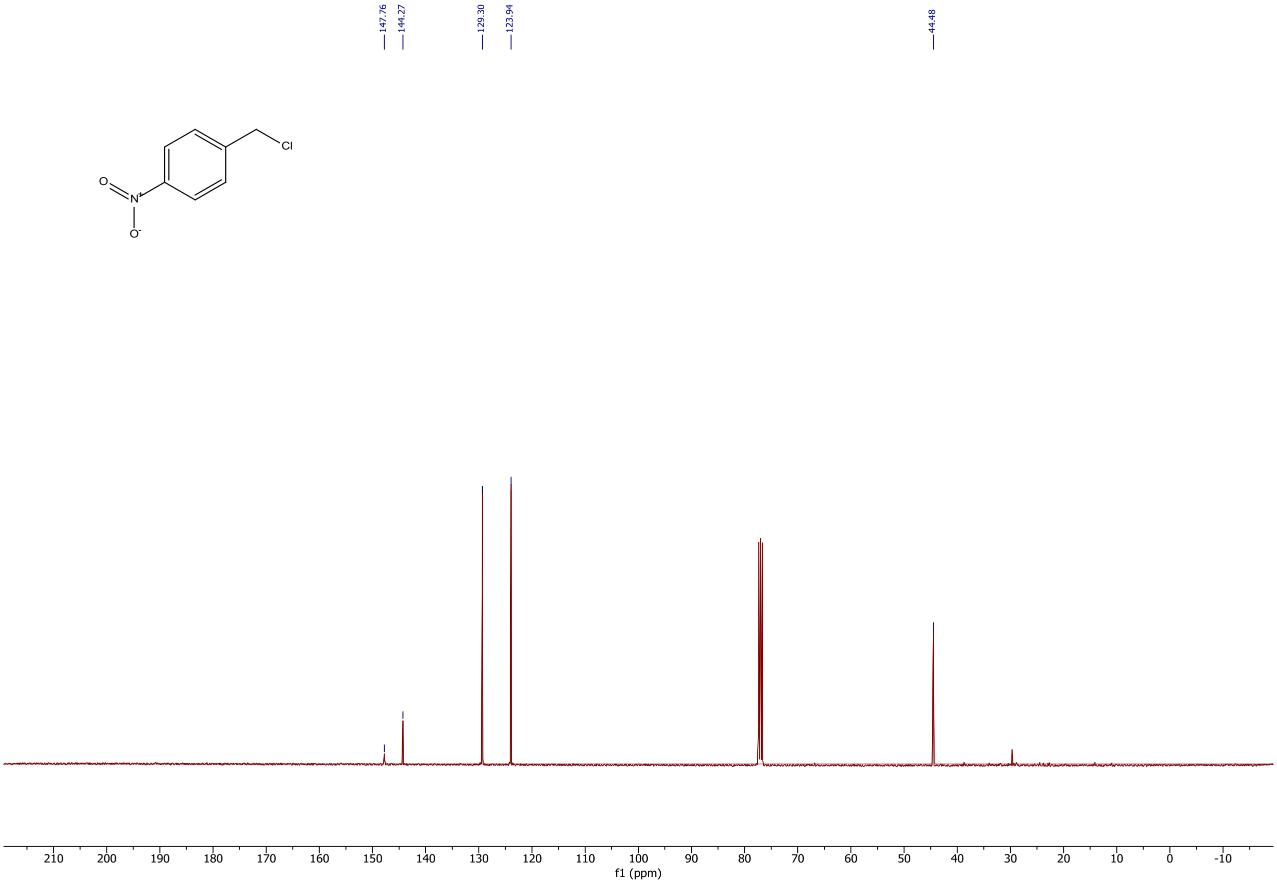

1-(chloromethyl)-2-nitrobenzene (3f)

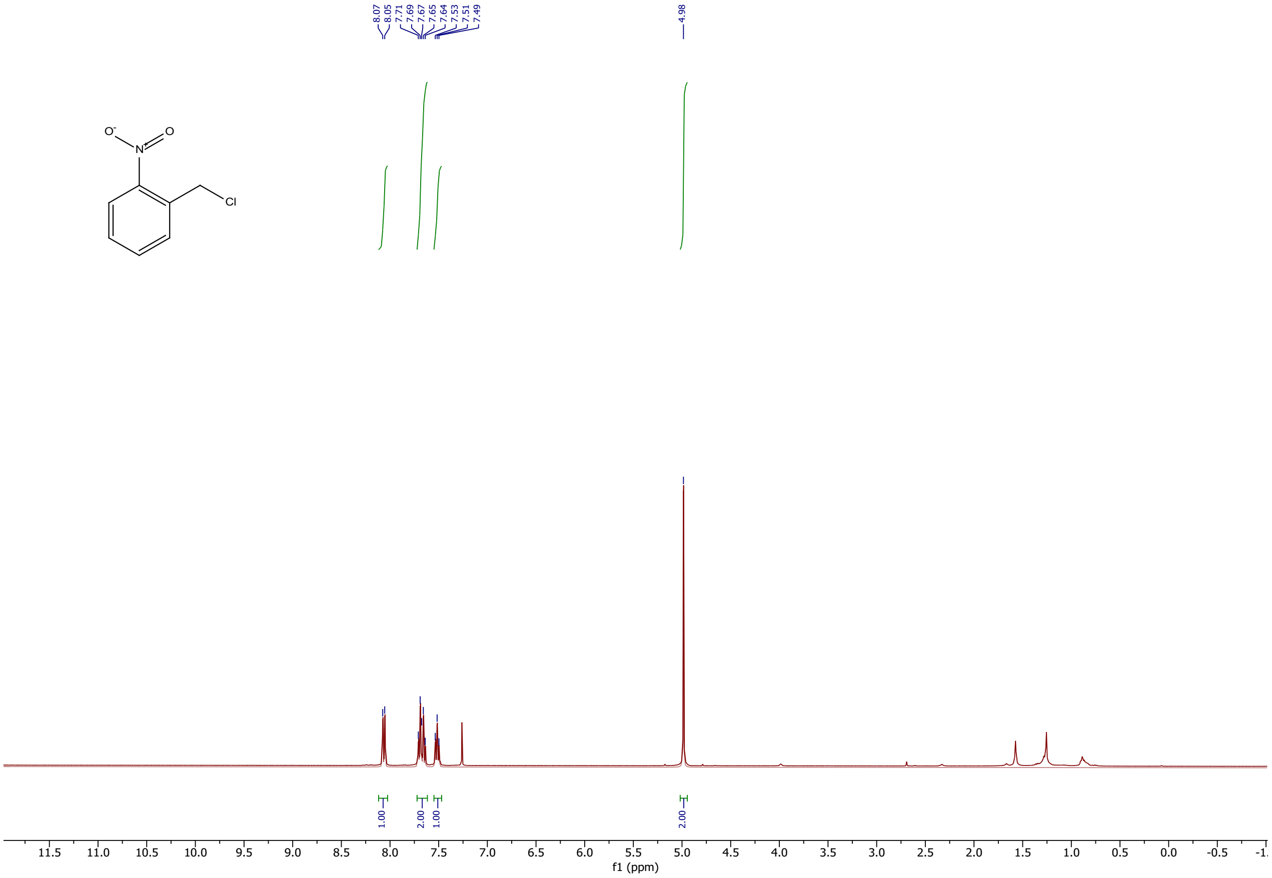

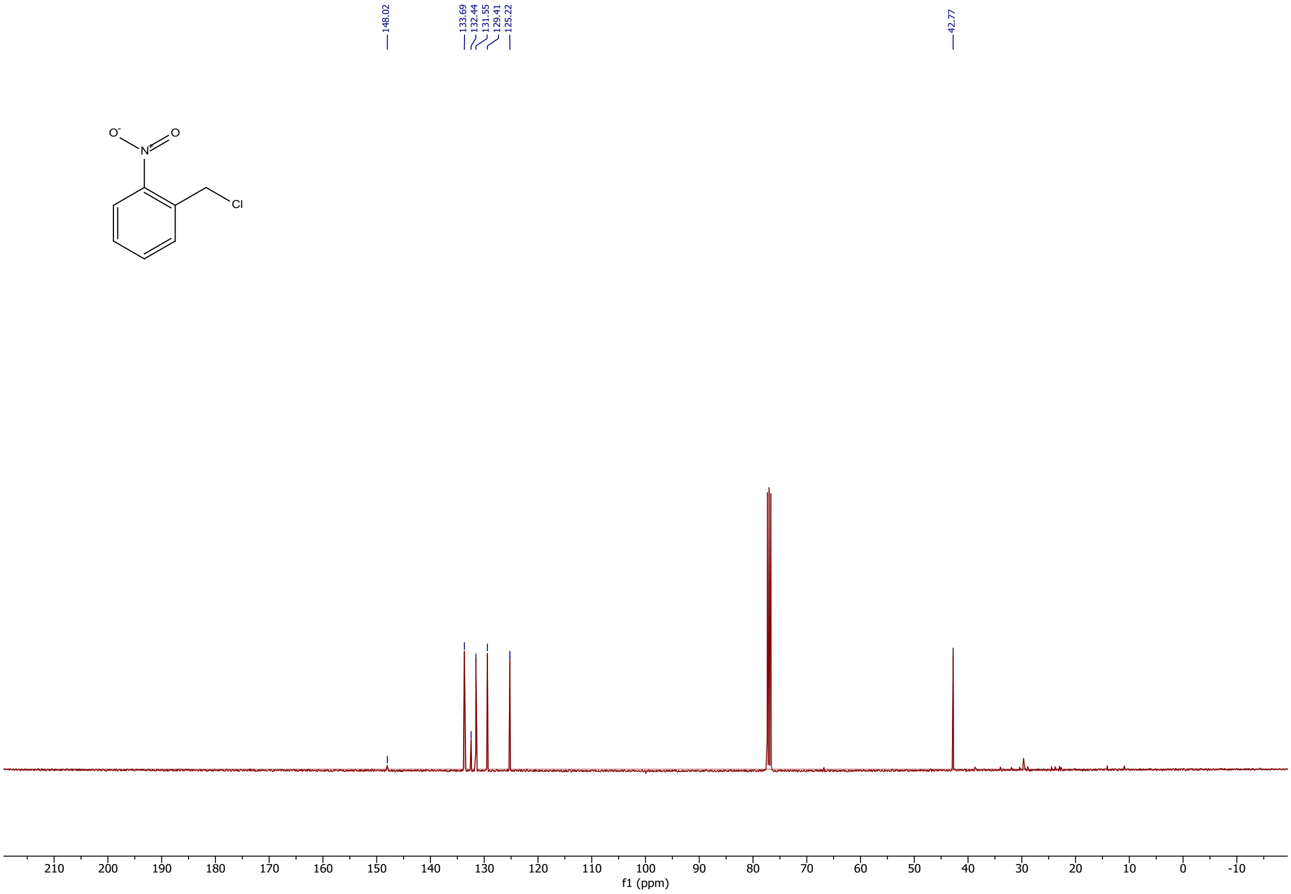

(4-(chloromethyl)phenyl)(phenyl)methanone (3g)

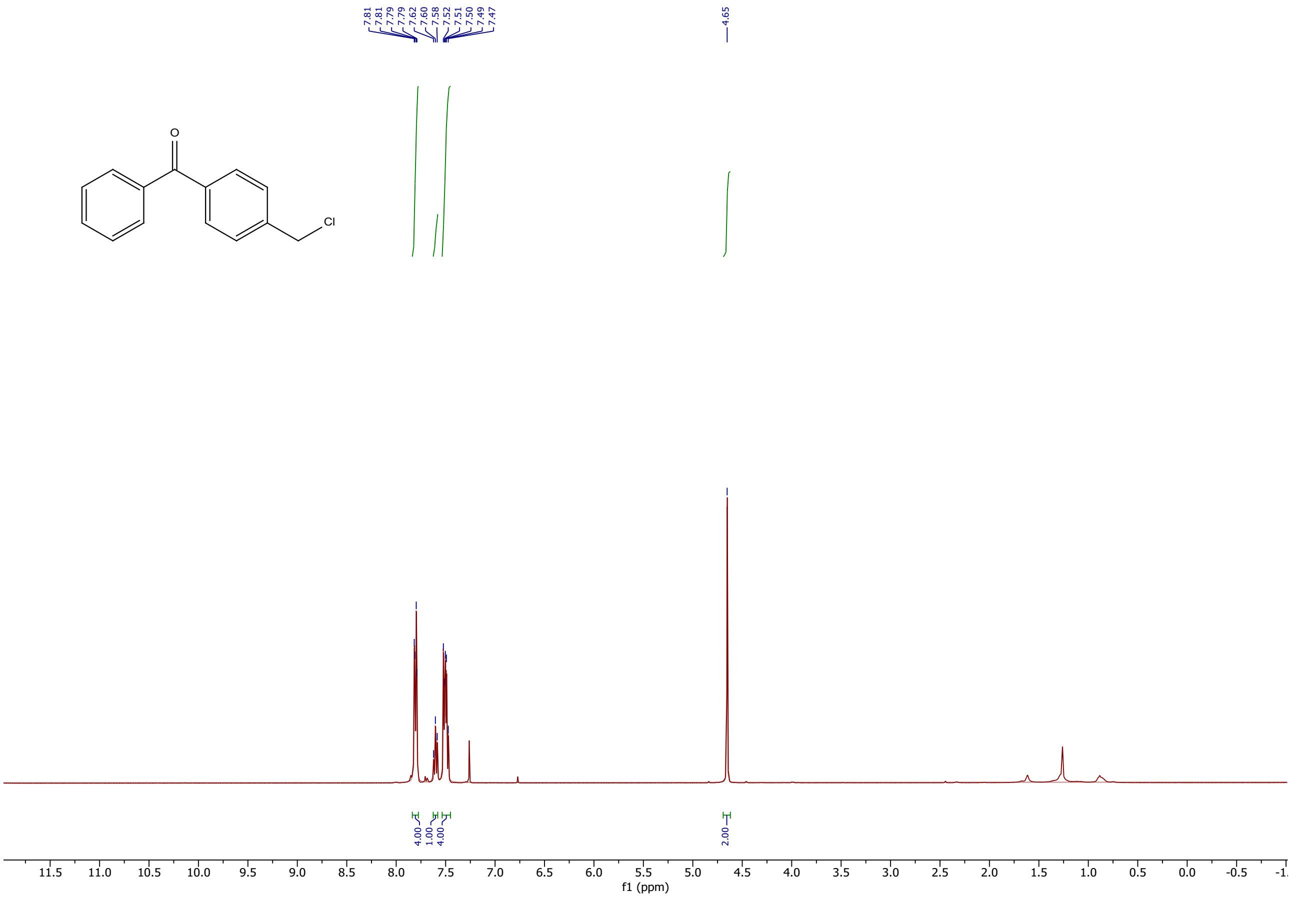

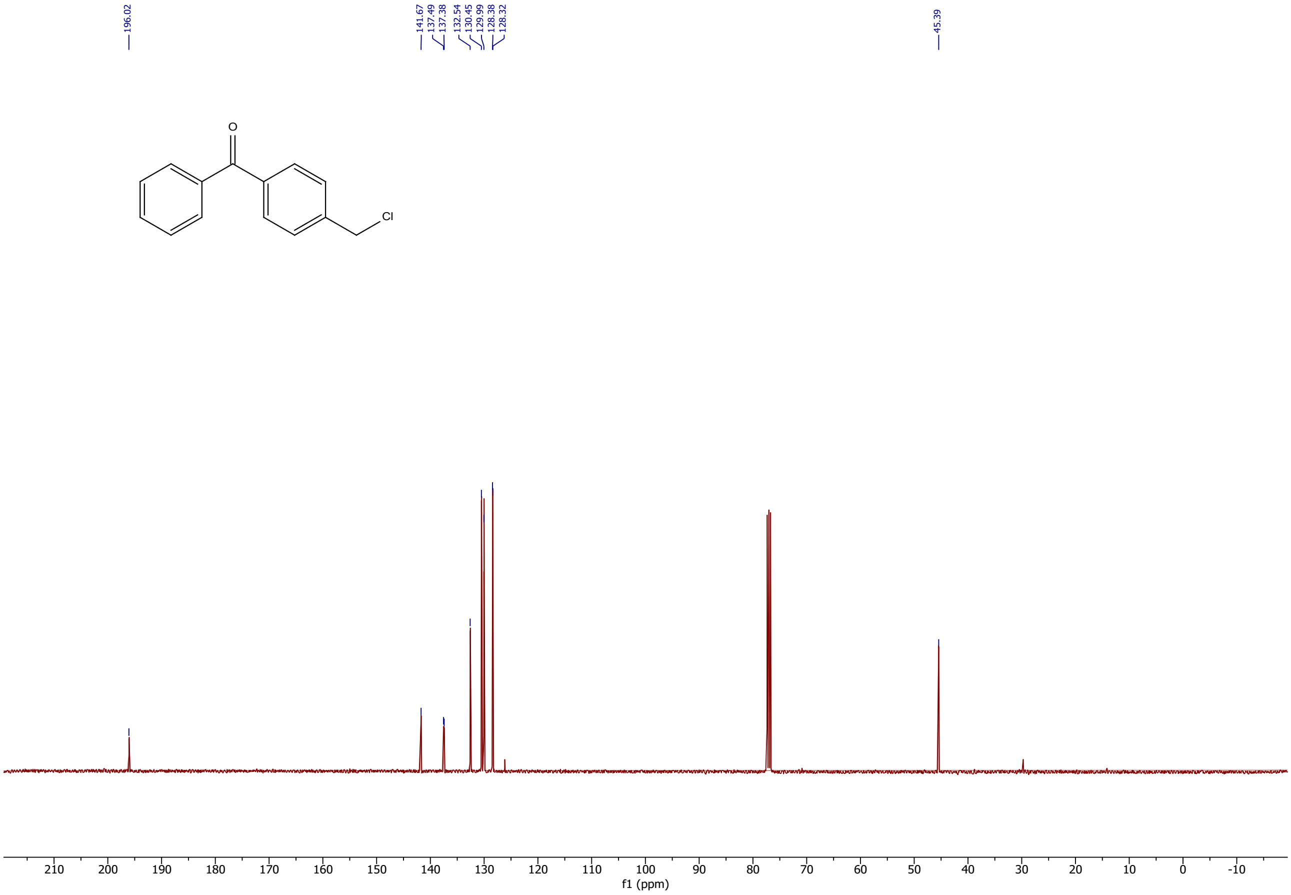

1-chloro-4-(chloromethyl)benzene (3h)

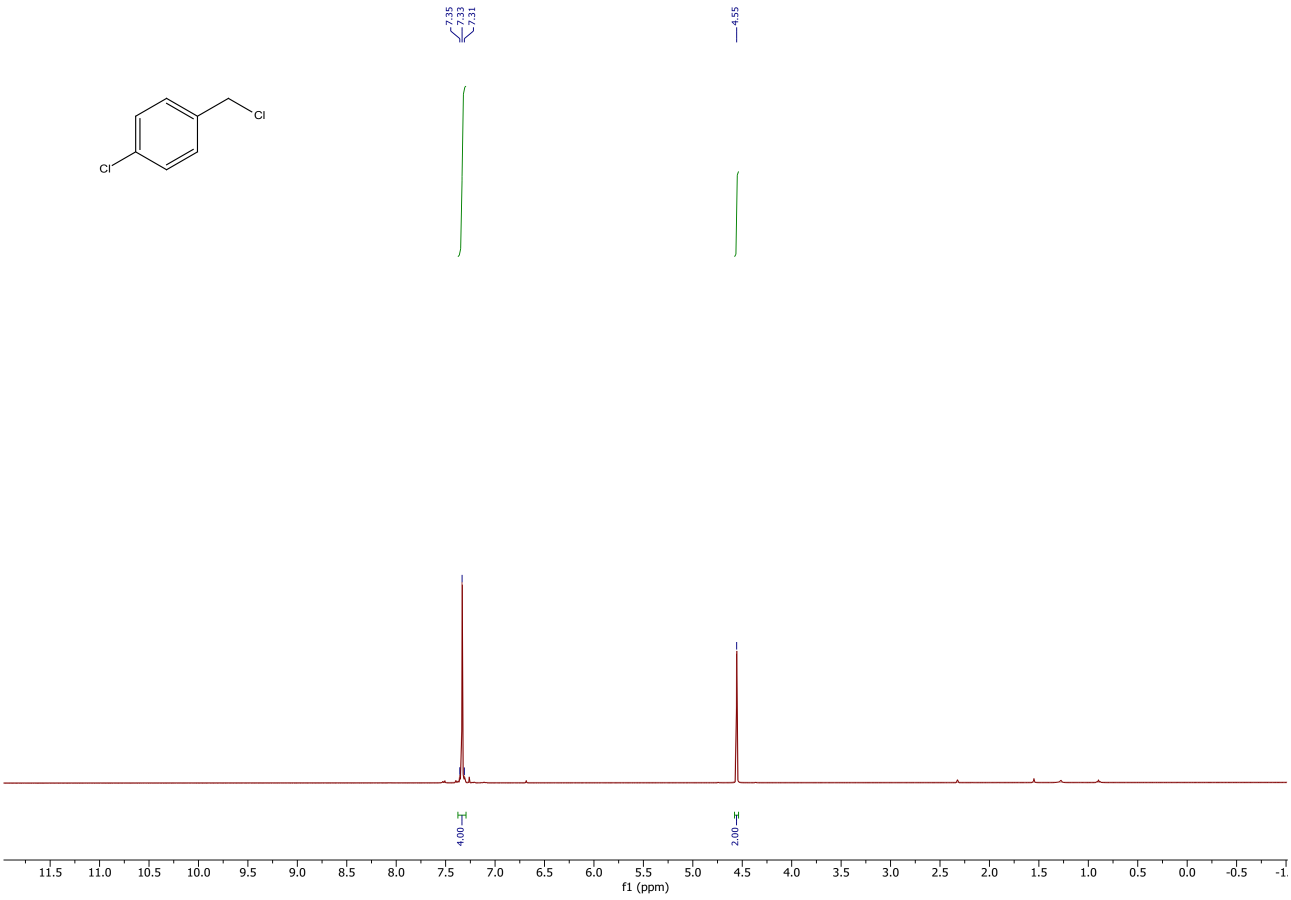

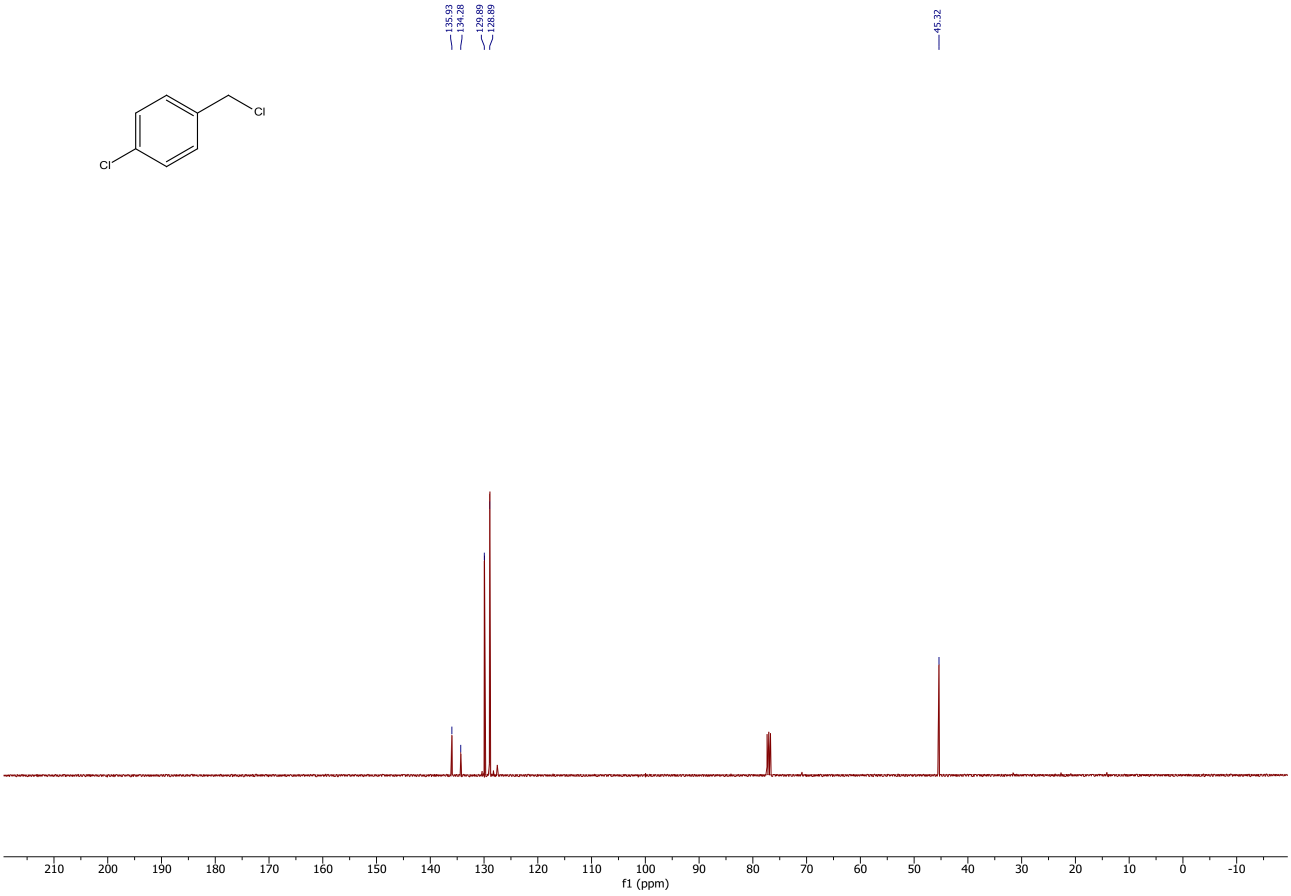

1-chloro-2-(chloromethyl)benzene (3i)

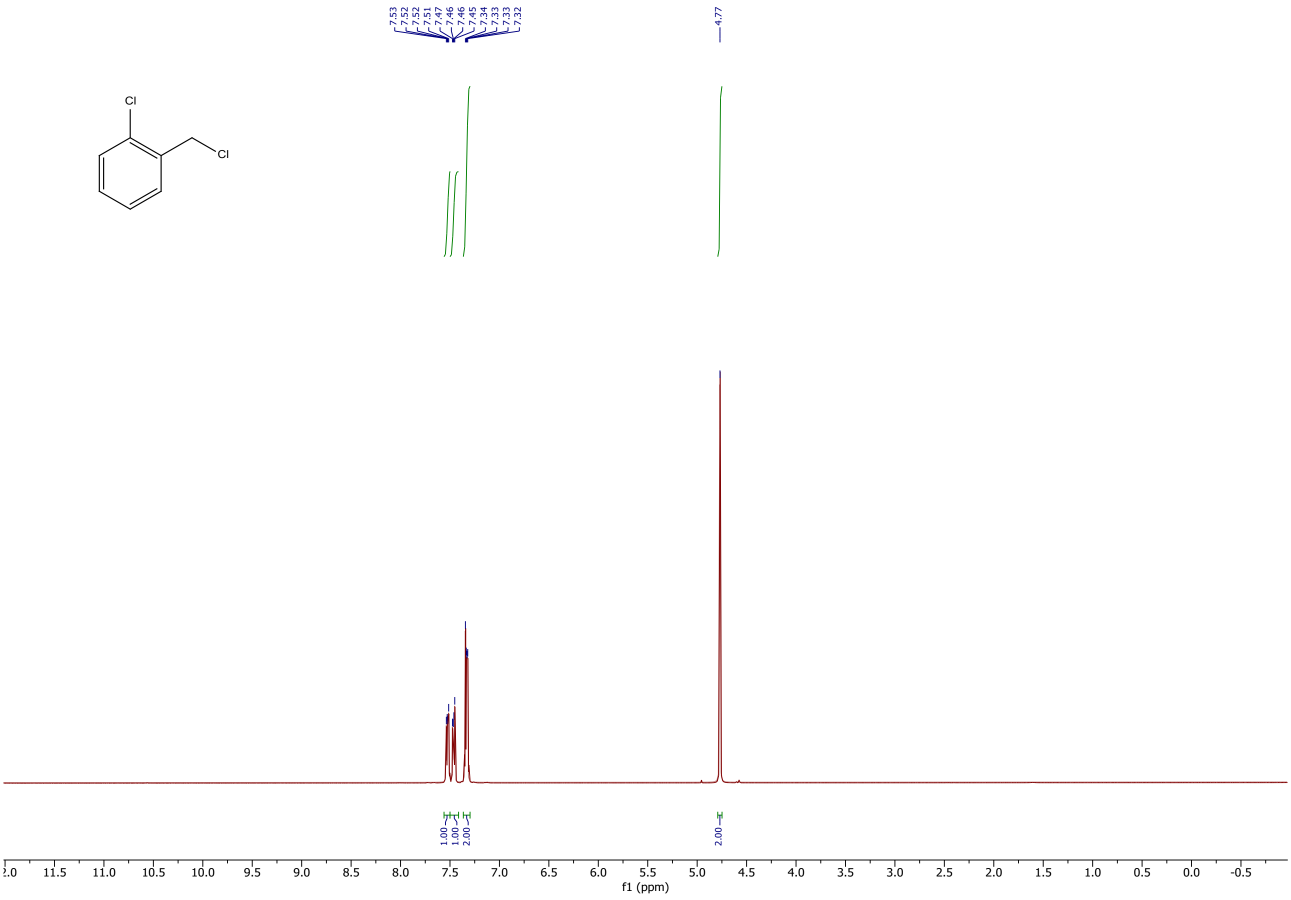

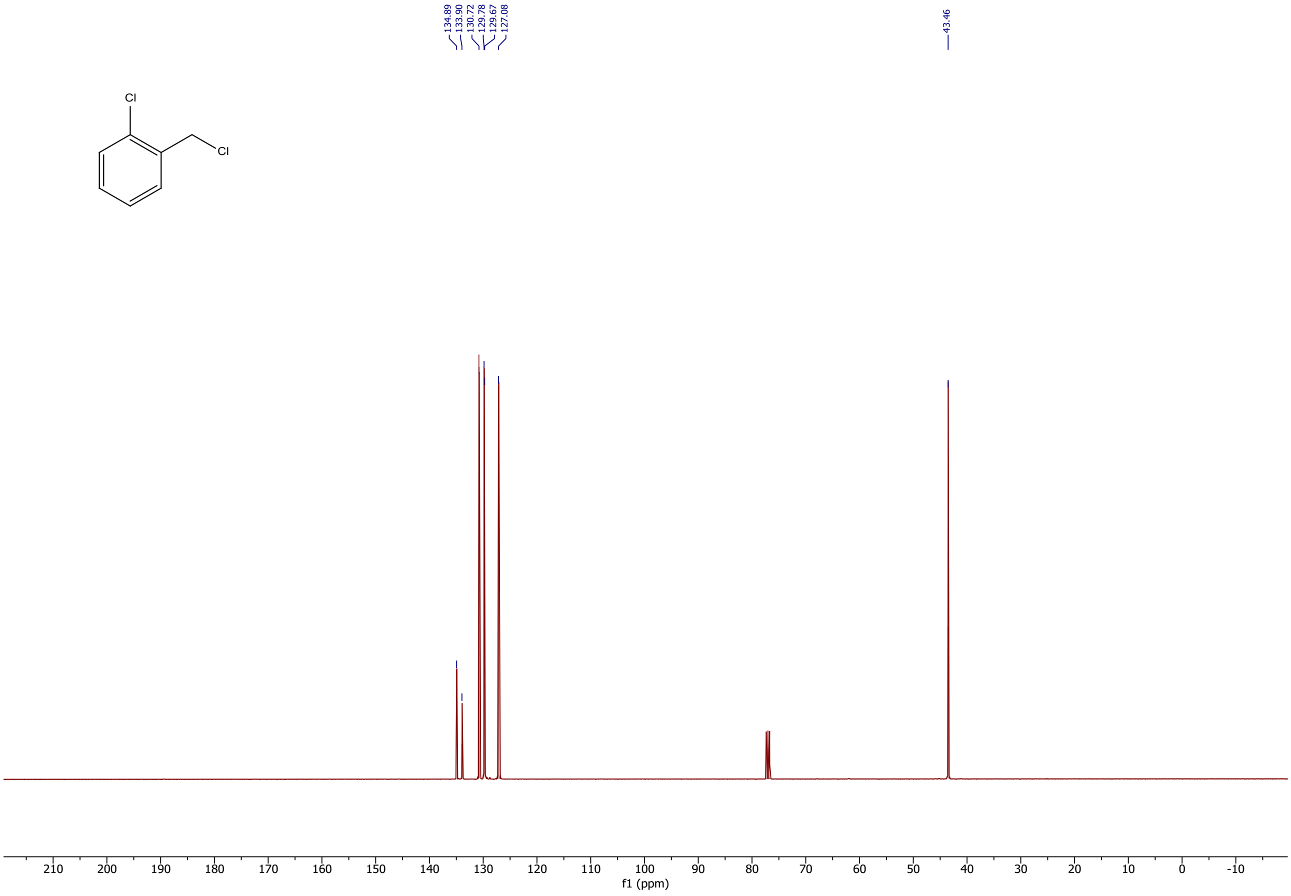

1,3-dichloro-2-(chloromethyl)benzene (3j)

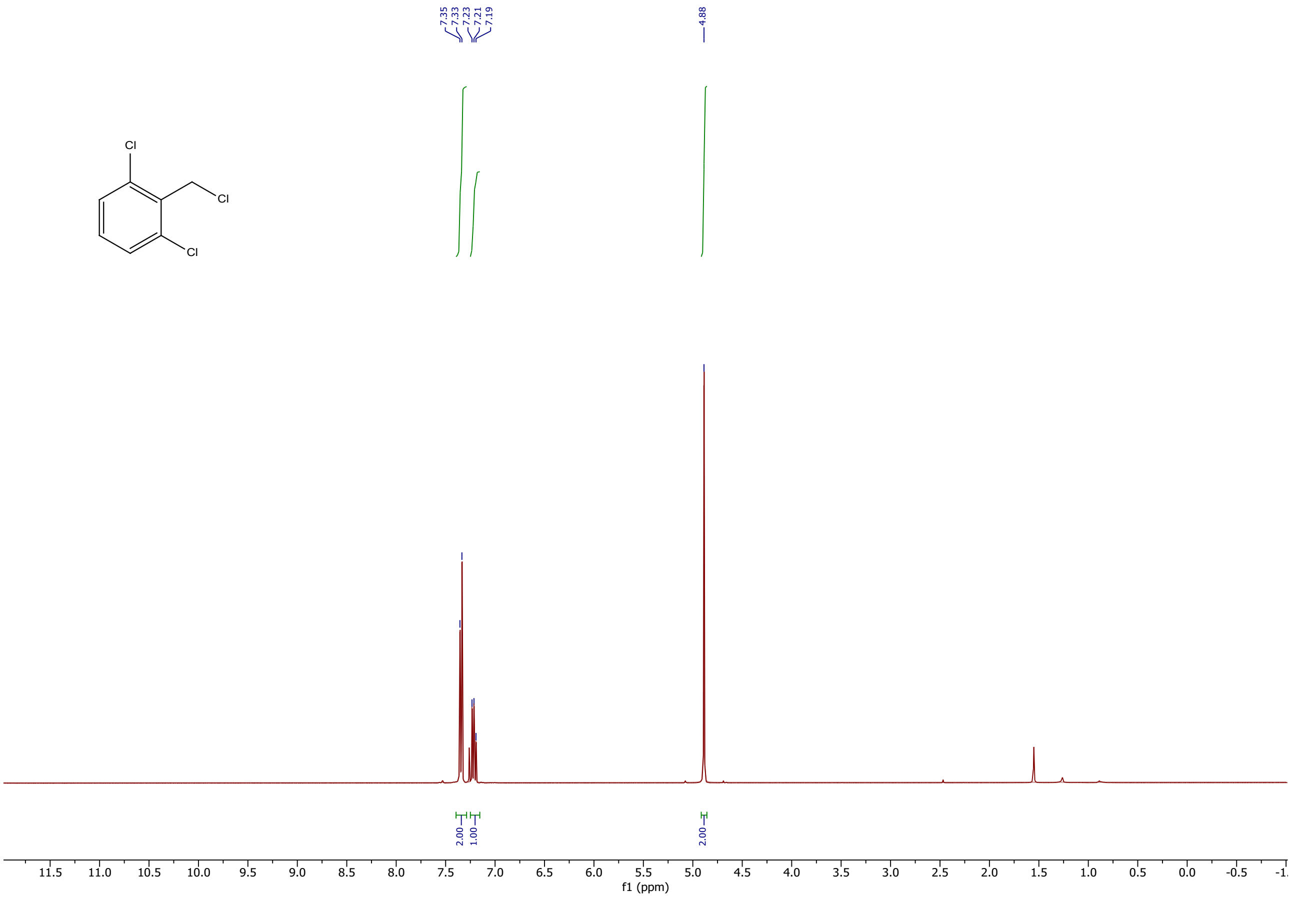

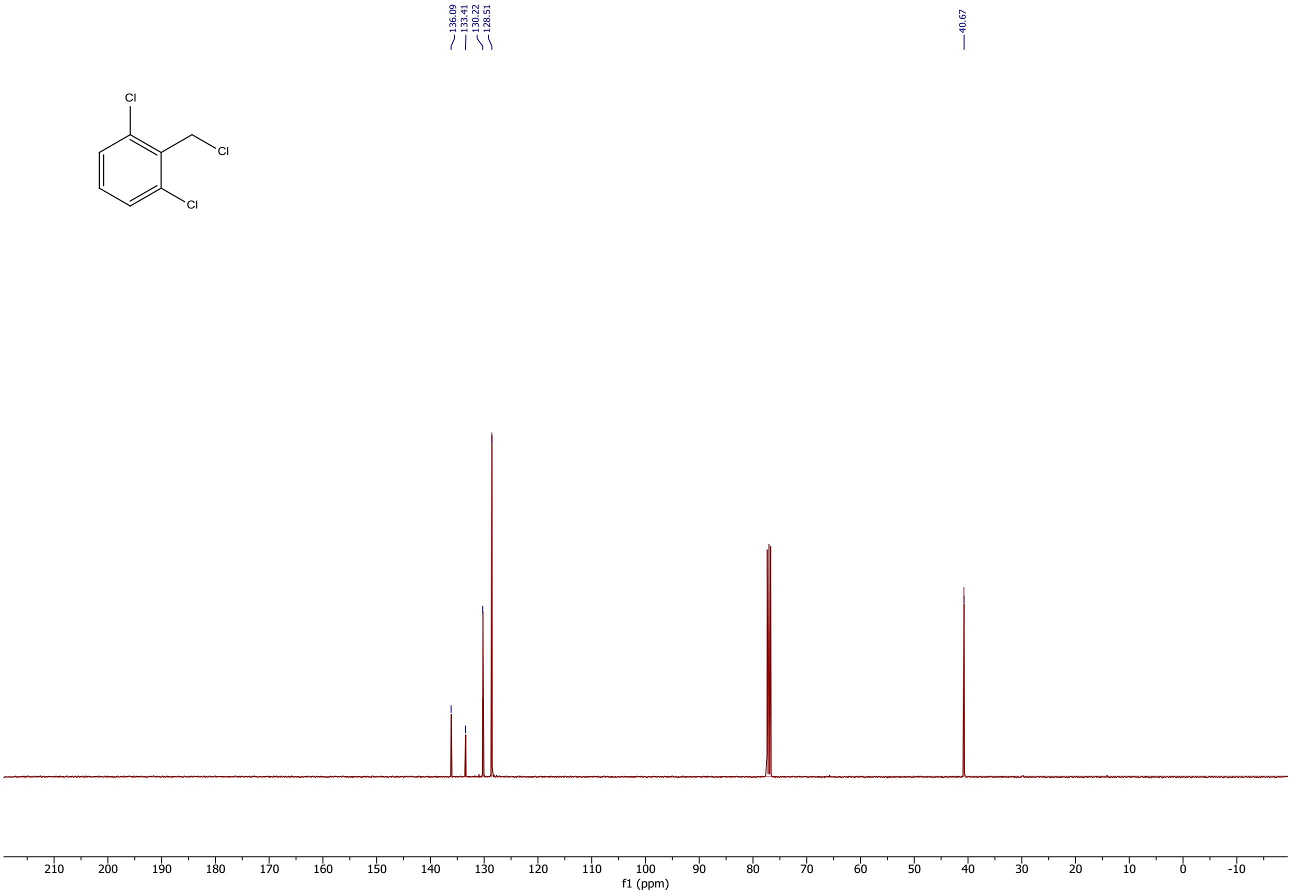

1-(chloromethyl)-4-fluorobenzene (3k)

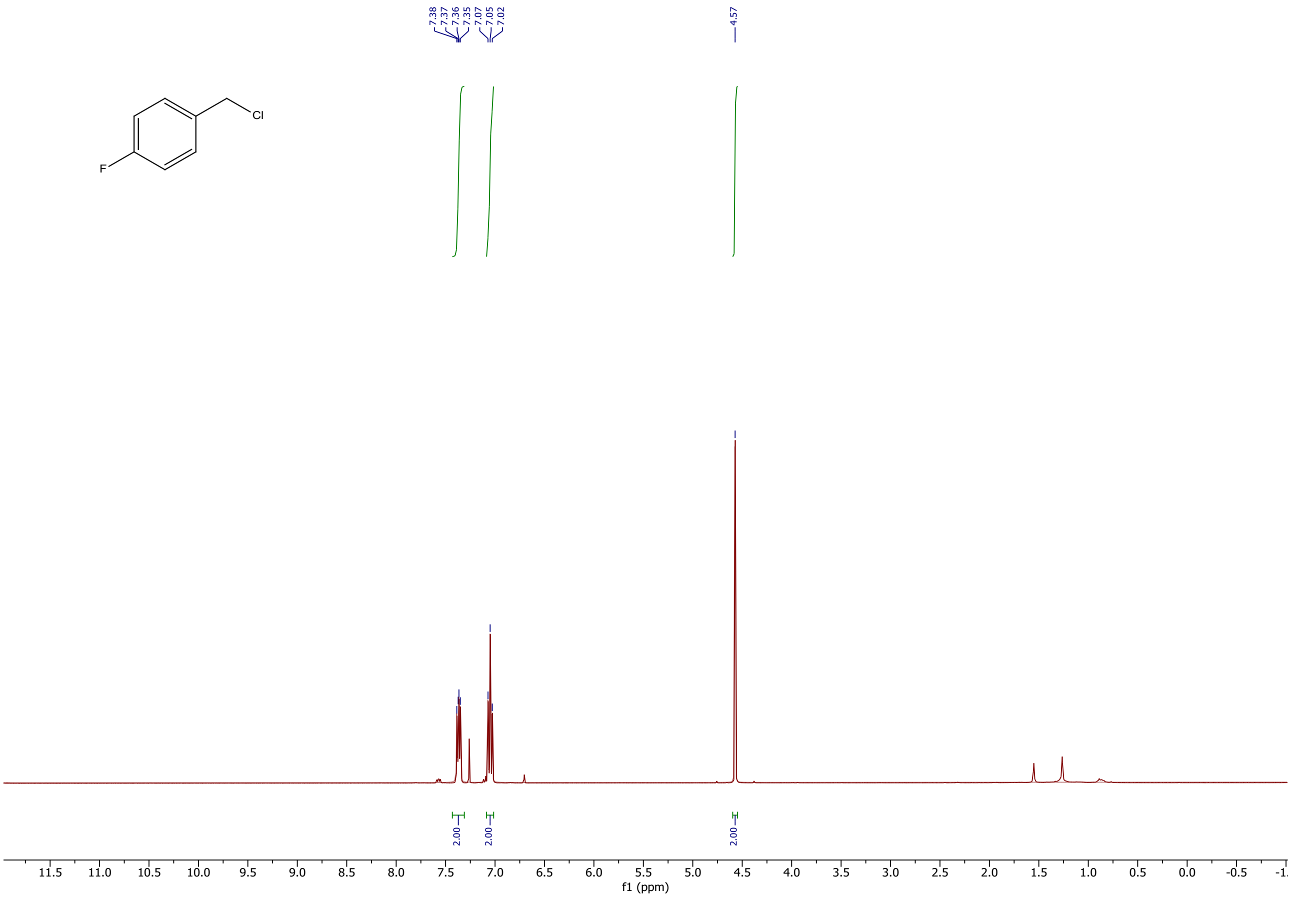

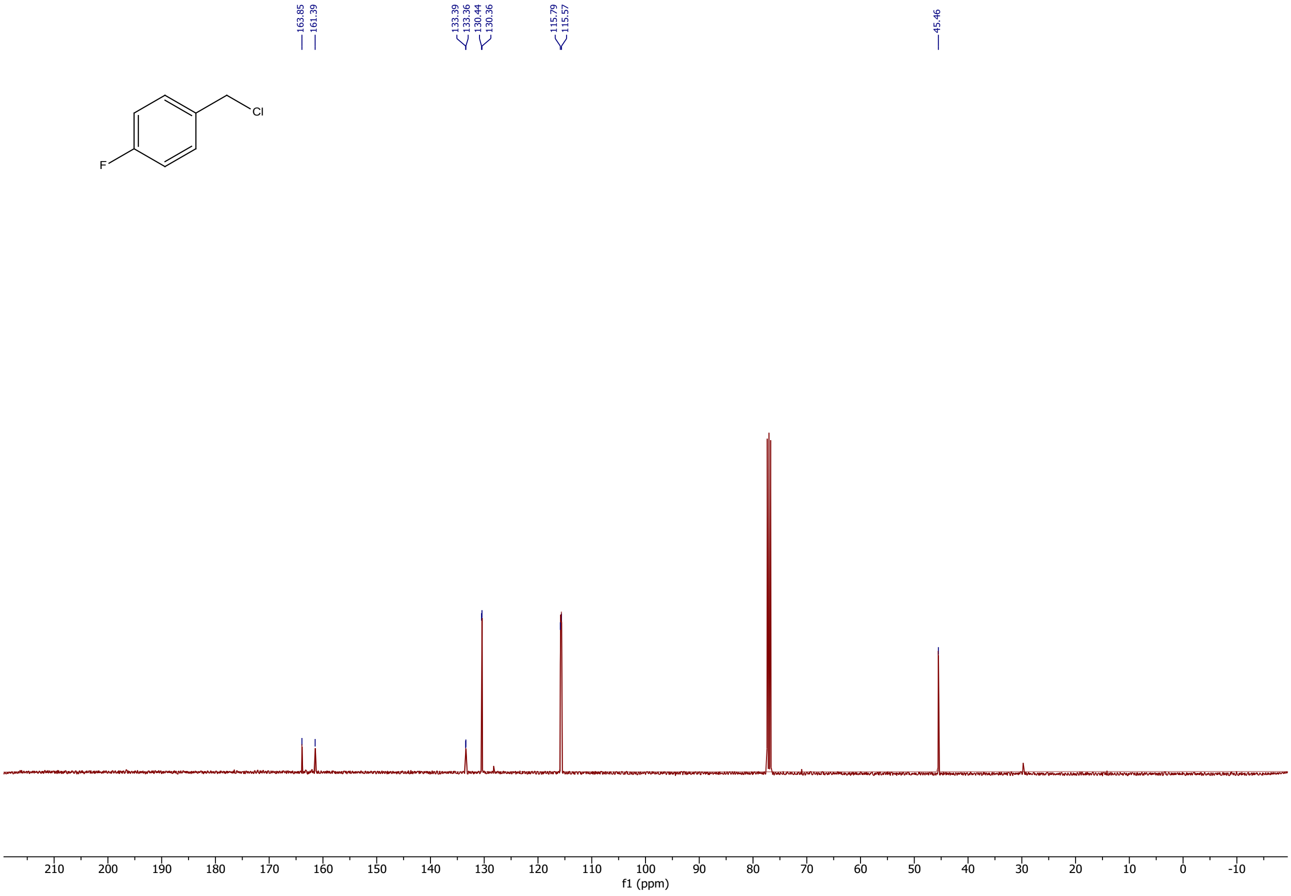

1-(chloromethyl)-4-methylbenzene (3l)

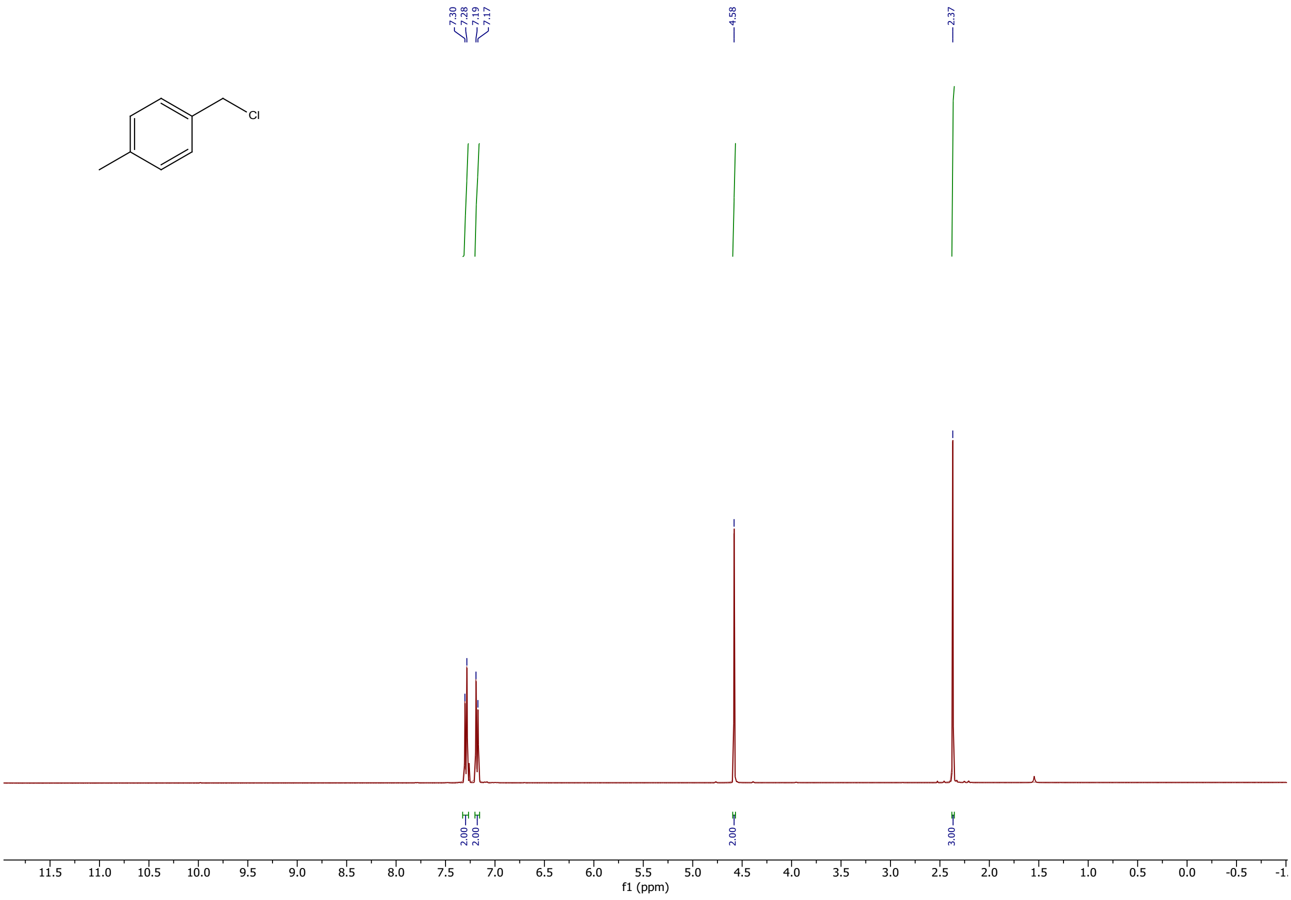

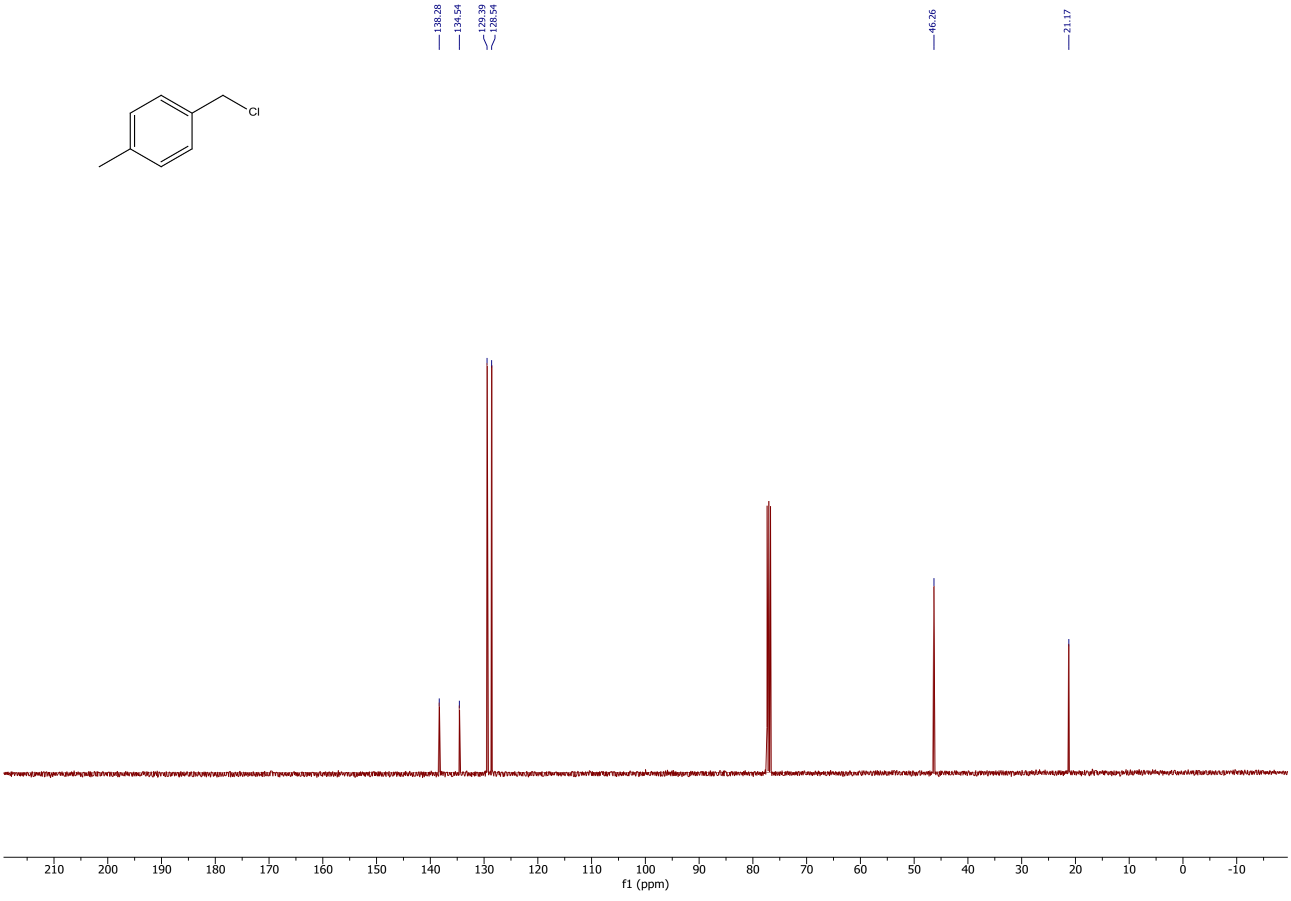

4-(chloromethyl)-1,1'-biphenyl (3m)

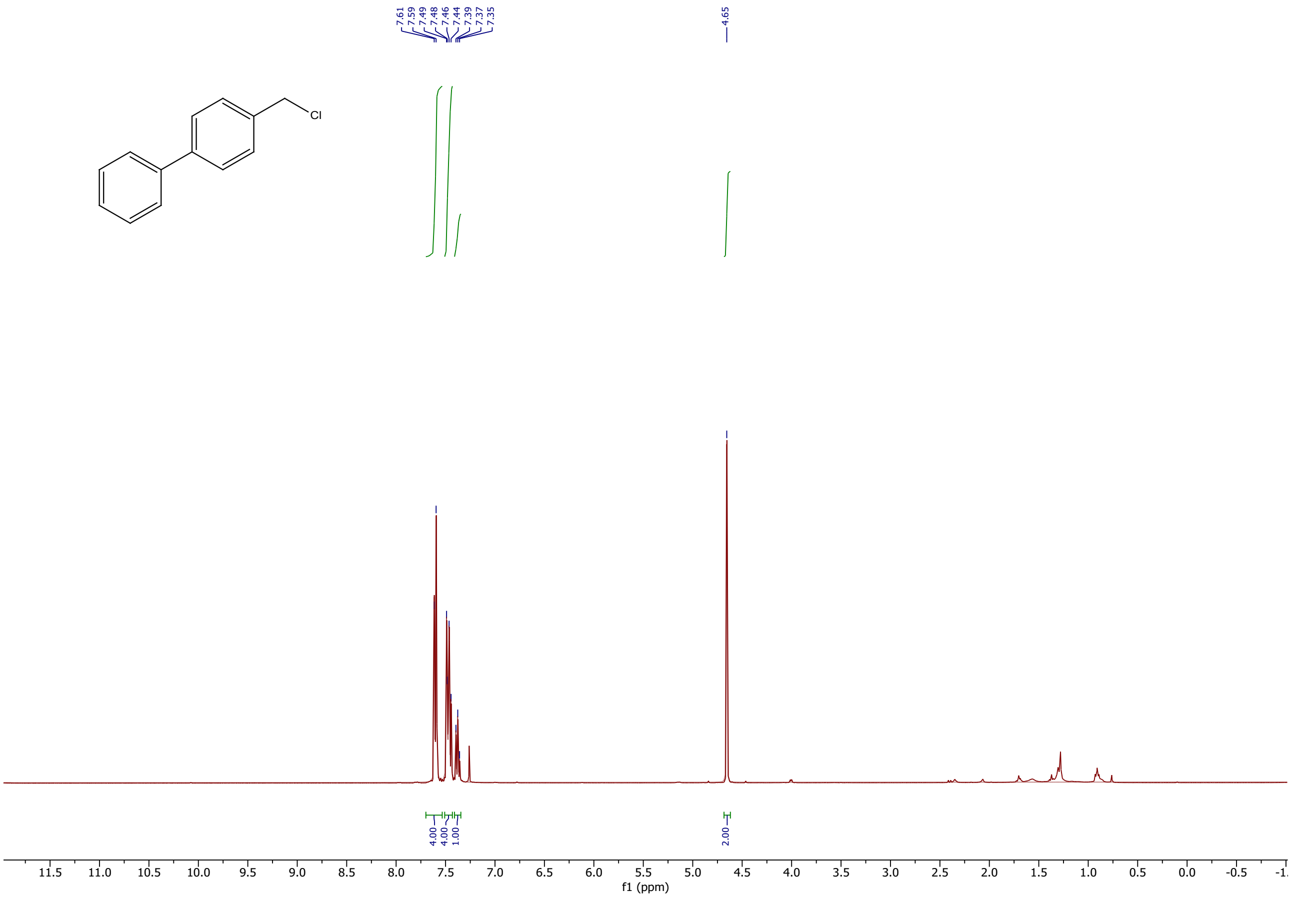

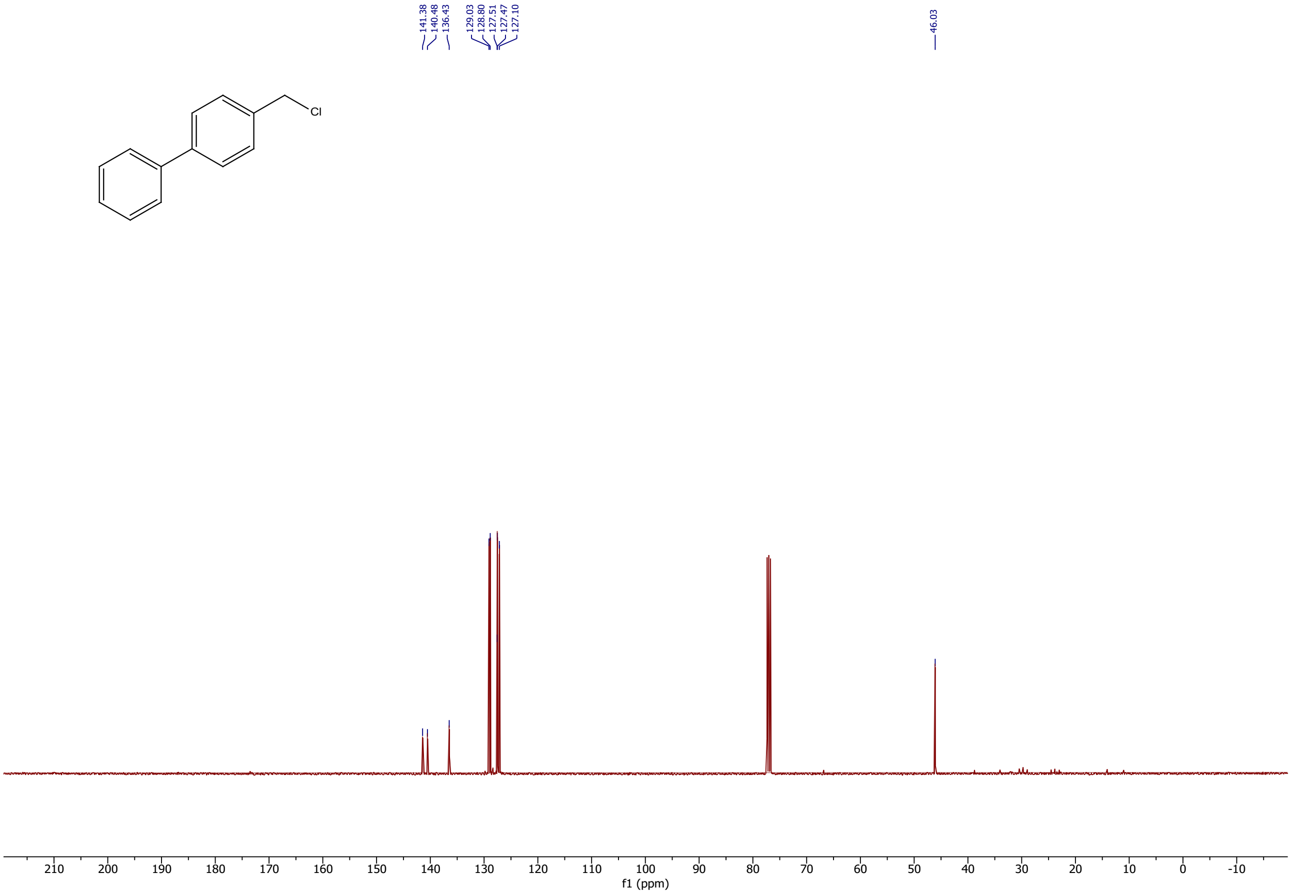

1-(*tert*-butyl)-4-(chloromethyl)benzene (3n)

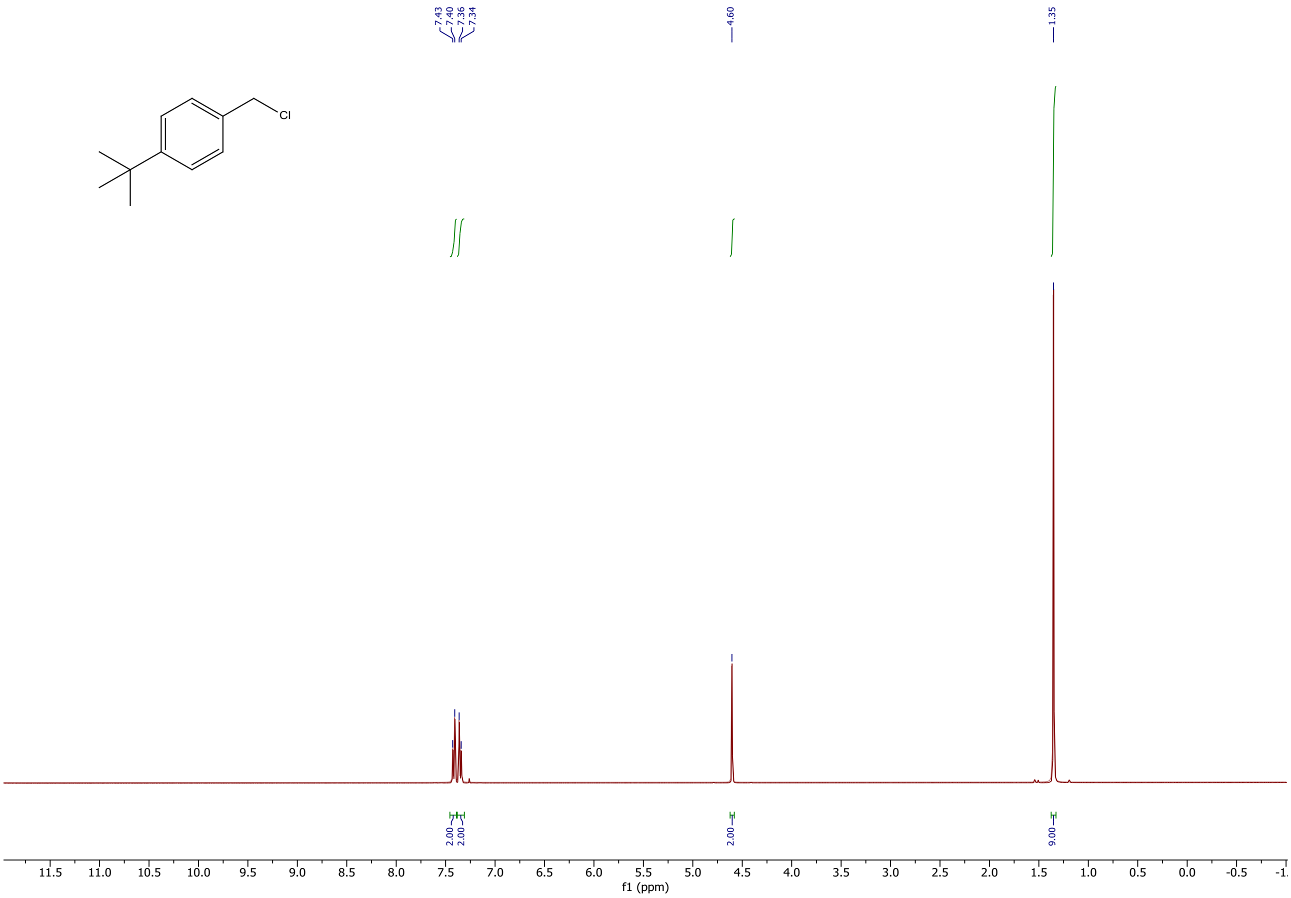

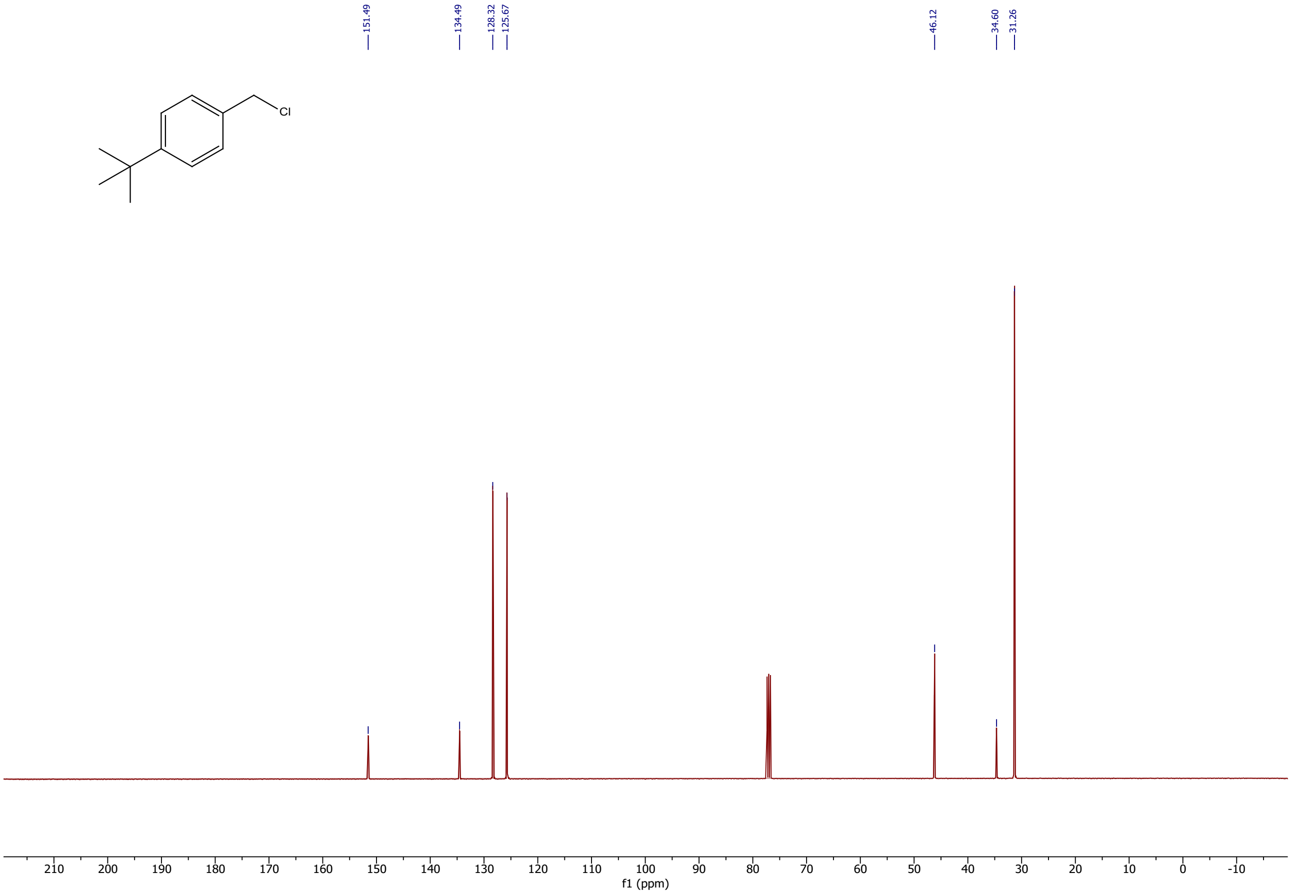

(1-chloroethyl)benzene (3o)

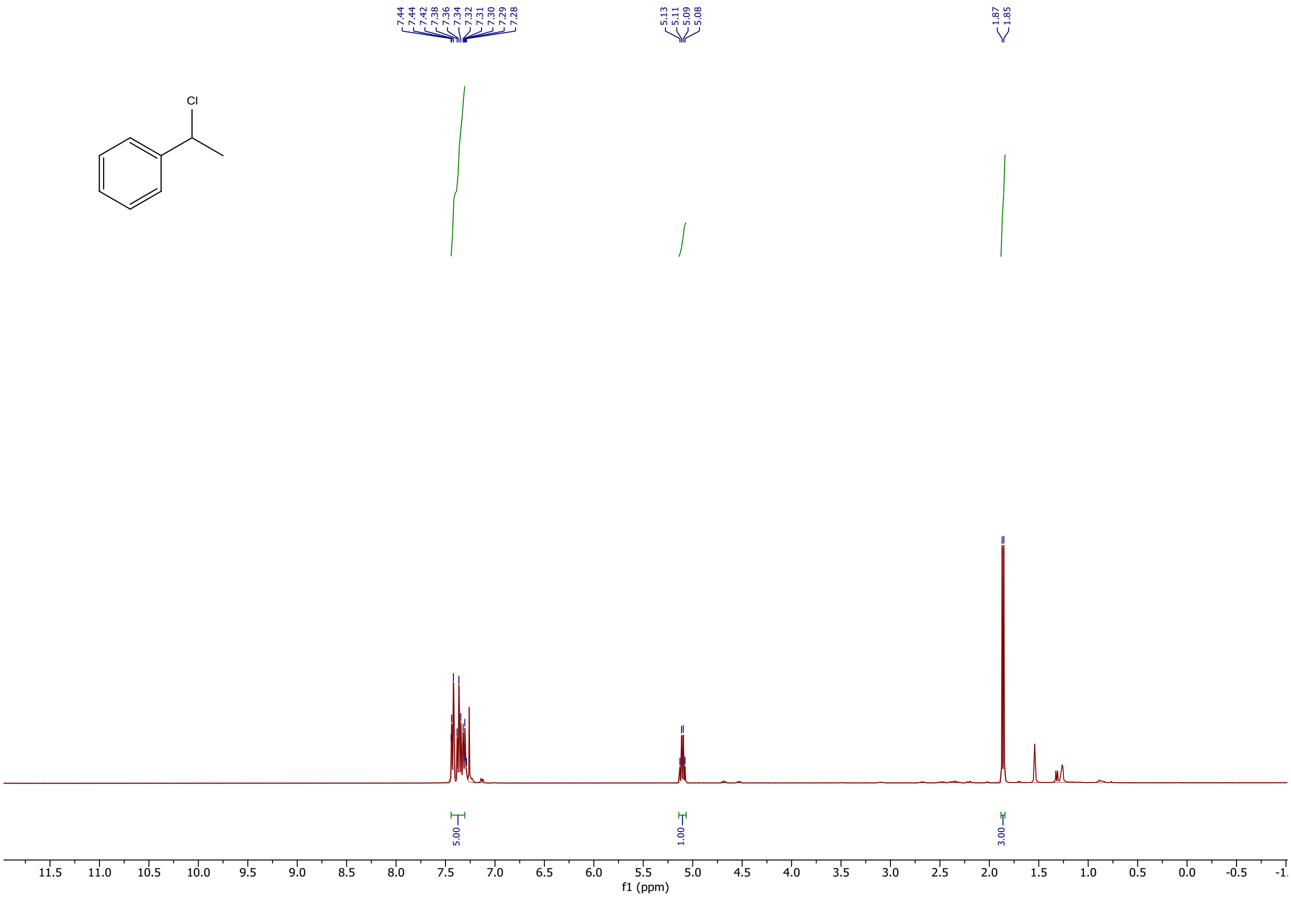

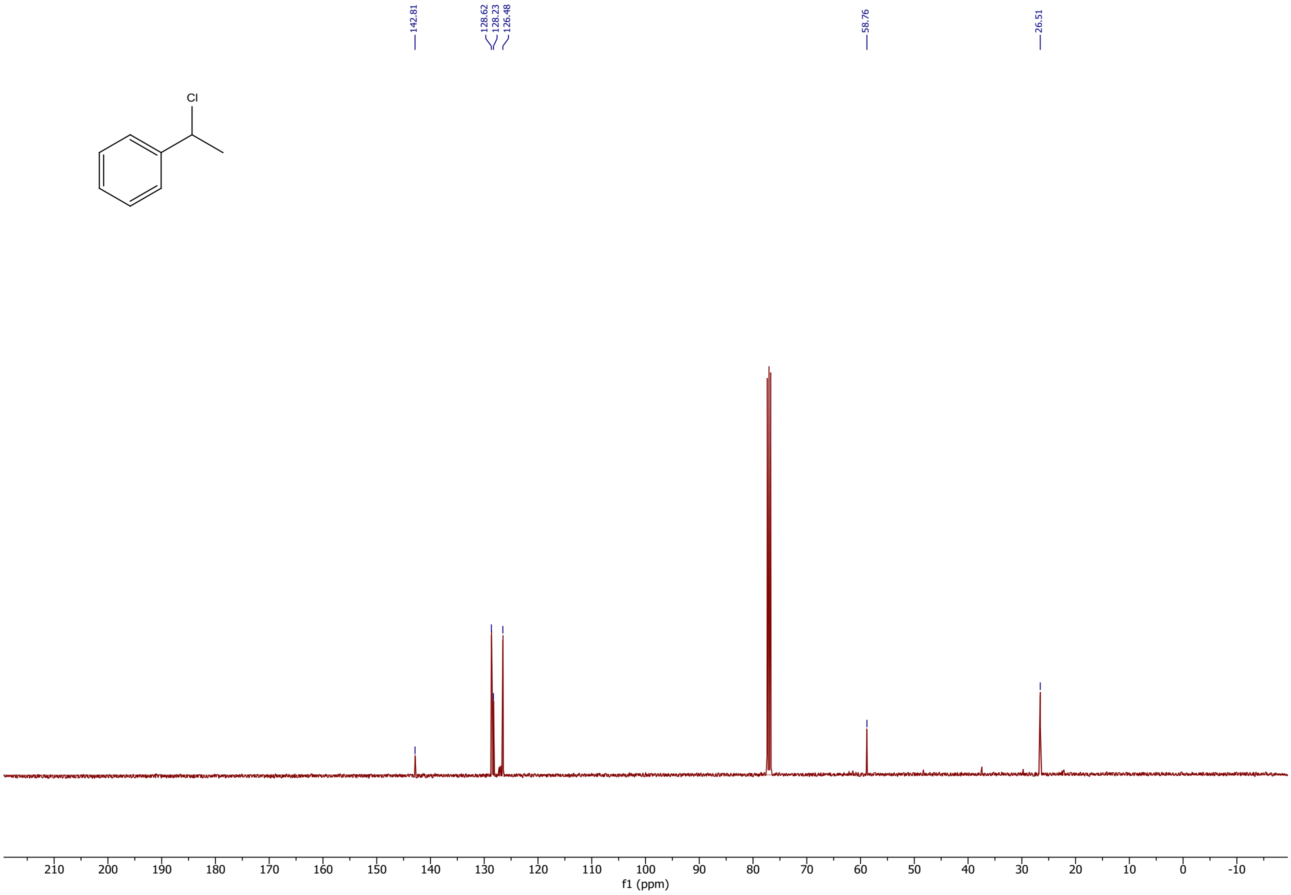

Supplement: Supplementary file 1 [file molecules-30-00312-s001.zip › molecules-3373294-supplementary.pdf]
